# TTLOC: A Tn5 transposase-based approach to localize T-DNA integration sites

**DOI:** 10.1093/plphys/kiaf102

**Published:** 2025-03-25

**Authors:** Xiao-Yuan Tao, Shou-Li Feng, Xin-Jia Li, Yan-Jun Li, Wei Wang, Matthew Gilliham, Zhong-Hua Chen, Sheng-Chun Xu

**Affiliations:** Biotechnology Institute, Xianghu Laboratory, Hangzhou 311231, China; Biotechnology Institute, Xianghu Laboratory, Hangzhou 311231, China; Biotechnology Institute, Xianghu Laboratory, Hangzhou 311231, China; Biotechnology Institute, Xianghu Laboratory, Hangzhou 311231, China; National Key Laboratory of Crop Genetics & Germplasm Enhancement and Utilization, College of Agriculture, Nanjing Agricultural University/Zhongshan Biological Breeding Laboratory/Collaborative Innovation Center for Modern Crop Production (CIC-MCP), Nanjing, Jiangsu 210095, China; ARC Centre of Excellence Plants for Space, School of Agriculture, Food and Wine, Waite Research Precinct, University of Adelaide, Glen Osmond, SA 5064, Australia; School of Science, Western Sydney University, Penrith, NSW 2751, Australia; Biotechnology Institute, Xianghu Laboratory, Hangzhou 311231, China; Institute of Digital Agriculture, Zhejiang Academy of Agricultural Sciences, Hangzhou 310021, China

## Abstract

Thermal asymmetric interlaced-polymerase chain reaction-based and whole-genome sequencing-based T-DNA localization approaches have been developed for the recovery of T-DNA integration sites (TISs). Nevertheless, a low-cost and high-throughput technique for the detection of TISs, which would facilitate the identification of genetically engineered plants, is in high demand for rapid crop breeding and plant synthetic biology. Here, we present Tn5 transposase-based T-DNA integration site localization (TTLOC), a Tn5-based approach for TIS localization. TTLOC employs specialized adaptor-assembled Tn5 transposases for genomic DNA tagmentation. TTLOC library construction is straightforward, involving only six steps that requires two and a half hours to complete. The resulting pooled library is compatible with next-generation sequencing, which enables high-throughput determination. We demonstrate the ability of TTLOC to recover 95 non-redundant TISs from 65 transgenic Arabidopsis (*Arabidopsis thaliana*) lines, and 37 non-redundant TISs from the genomes of transgenic rice (*Oryza sativa*), soybean (*Glycine max*), tomato (*Solanum lycopersicum*), potato (*Solanum tuberosum*), and from the large hexaploid wheat (*Triticum aestivum*) genome. TTLOC is a cost-effective method, as 1 to 2 Gb of raw data for each multiplexing library are sufficient for efficient TIS calling, independent of the genome size. Our results establish TTLOC as a promising strategy for evaluation of genome engineered plants and for selecting genome safe harbors for trait stacking in crop breeding and plant synthetic biology.

## Teaser

TTLOC is a Tn5 transposase-based tool for evaluating genetically engineered plants, with potential applications in crop breeding and plant synthetic biology.

## Introduction

Plant genetic engineering, including transgenic and genome editing, for the rapid generation of engineered crops is promising to accelerate and broaden breeding programs (Chen and Liu 2023). Important targets of these technologies beyond herbicide and pesticide tolerance include high environmental resilience, increase of productivity, improvement of crop nutritional quality, and resistance to disease (Schauer et al. 2006; Mao et al. 2007; Chen et al. 2021). The efficient generation of transgenic or gene-edited plants relies on transformation and regeneration. Most frequently used plant transformation strategies include Agrobacterium-mediated transformation, biolistic particle bombardment, and gene delivery based on various nanoparticles and virus-based RNA delivery systems (de Groot et al. 1998; Mäenpää et al. 1999; Sigoillot et al. 2012), in which Agrobacterium-mediated transformation is a cost-effective and efficient gene delivery system, which can transfer large fragments of DNA into plant chromosomes, remains the leading choice for plant transformation (Azizi-Dargahlou and Pouresmaeil 2024). Moreover, T-DNA transfer and stable transformation are also important for the delivery of the CRISPR-Cas system for efficient genome-editing in plants (Liu et al. 2023). While bringing immense opportunities for sustainable agriculture, the application of genetically modified organisms (GMOs) is associated with regulatory and intellectual property challenges to the environment (Kay and Van den Eede 2001; Lang et al. 2007; Weimer 2015; Egelie et al. 2016), which raises the importance of safe assessment of genetically engineered plants.

Besides the safety assessment of GMOs to the environment, the GMOs generated by Agrobacterium-mediated transformation also need safety assessment to the plant itself. Following Agrobacterium inoculation, a single-strand form of T-DNA (T-strands) likely forms complexes with virulence effector and plant proteins that traffic through the cytoplasm to enter the nucleus (Gelvin 2017). T-strands will be randomly integrated into plant chromosomes facilitating transgenic expression, a process known as stable transformation. T-DNA integration is rarely a clean event, it usually causes genomic changes at TISs, including deletion, duplication, filler DNA sequences, binary vector backbone sequences, and chromosomal rearrangement, leading to disruption of the stable expression of internal genes (van Kregten et al. 2016; Nishizawa-Yokoi et al. 2021). Moreover, the configuration and genomic position of integrated T-DNA molecules can affect expression of the transgene. An ideal integration is that the single-copy T-DNA between left border (LB) or right border (RB) is inserted into the plant genome with accurate repair (van Kregten et al. 2016). However, it usually occurs with multiple copies of T-DNAs in tandem or inverted repeats and the concatenation of multiple ideal or truncated partial T-DNAs. Thus, the determination of T-DNA copies, T-DNA integration sites, and sequence changes of flanking regions is important for stable expression of genes and safety assessment of genetically engineered plants from the lab to the field.

Polymerase chain reaction (PCR)-based technologies (e.g. thermal asymmetric interlaced PCR [TAIL-PCR], Adapter-PCR, inverse PCR [iPCR]) were developed, optimized, and widely used for identification of T-DNA integration sites (Liu and Whittier 1995; Liu and Chen 2007; O'Malley et al. 2007; Kim et al. 2011; Tan et al. 2019). However, its procedure is relatively complicated due to the design of primers and the time-consuming multiple rounds of PCR operation. Other method involves the hybridization and capture of sequences containing T-DNA borders using biotinylated oligos and subjected to Illumina-Miseq (Reynoso et al. 2018). In comparison, whole-genome sequencing (WGS)-based procedures including TDNAscan (Chen et al. 2022) and T-LOC (Li et al. 2022) are straightforward for T-DNA integration sites (TISs) analysis. A diversified pattern of T-DNA integration was established by WGS-based T-LOC procedure for transgenic rice (Li et al. 2022). However, to obtain the required coverage (30×) of sequencing depth, the cost for WGS for each sample will dramatically increase, especially for large polyploid genomes such as cotton and wheat. Thus, advancing approaches that efficiently recover TISs for transgenic plants with large genome is of high importance to plant researchers and molecular breeders.

In this study, we aimed to establish a concise method for TIS recovery in transgenic plants with low cost and high performance. Tn5 transposase assembles with adaptors which contain Illumina adapter sequences and a 19 bp recognition site mosaic end (ME) sequences has the ability to cut double-stranded DNA in vitro and paste adaptor sequences to both 5′ end of DNA fragments for the following next-generation sequencing (NGS) library construction (Picelli et al. 2014). Mechanistically, by capitalizing on this “cut-and-paste” activity of Tn5 transposase, the DNA fragments containing T-DNA and their flanking genomic sequences can be tagged with adaptor sequences following Tn5 tagmentation, which facilitates the development of a high-throughput approach for retrieving TISs. Here, we developed a Tn5-based T-DNA integration site localization (TTLOC) approach for determination of TISs. We first introduced the working principle and procedure for TTLOC and its subsequent bioinformatic approaches for TISs recovery. We then showed successful applications of TTLOC to determine the TISs in 65 independent transgenic Arabidopsis (*A. thaliana*) lines and in 22 lines of other plant species including rice (*O. sativa*), soybean (*G. max*), tomato (*S. lycopersicum*), potato (*S. tuberosum*), and wheat (*T. aestivum*). Finally, we compared TTLOC, TAIL-PCR, and the WGS-based T-LOC approaches regarding their procedure, cost, and performance. We conclude that TTLOC is an important tool for TISs recovery, representing a substantial contribution to screening and identification of GMOs.

## Results

### TTLOC is a Tn5 tagmentation and NGS-based procedure for the recovery of TISs

**Figure 1.**
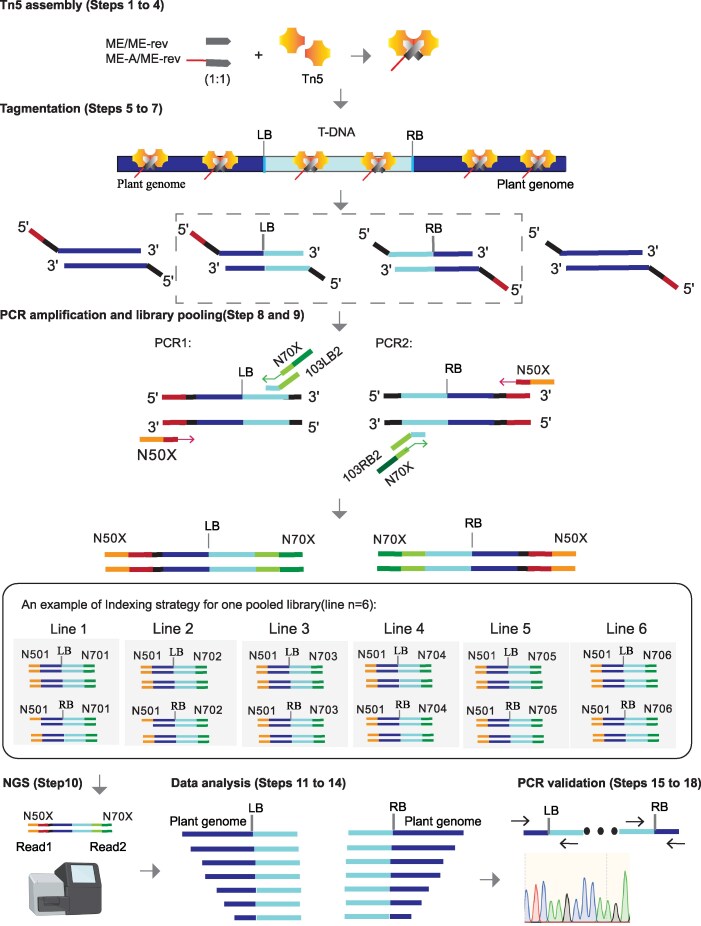
Workflow for TTLOC. Procedures of Tn5 transposase assembly, tagmentation, PCR amplification, library pooling, NGS sequencing, data analysis, and PCR validation were indicated. The corresponding step-to-step protocol is provided in the Supplementary Methods. Adaptor mix (ME/ME-rev:ME-A/ME-rev = 1:1)-assembled Tn5 was applied for tagmentation to generate DNA fragments tagged with adaptor sequences. After tagmentation, two separate PCRs were conducted to amplify flanking sequences of T-DNA left border (LB) or right border (RB) region. Three primers were added in each PCR, 2 of which are the shared P5 (N50X) and P7 (N70X) barcoding primers while the other one is the region-specific bridge primer for either LB (e.g. 103LB2) or RB (e.g. 103LB2) that has the P7 bridge sequences at its 5′ end. An example of indexing strategy for one pooled library was provided. PCR products of 6 independent lines with distinguishable barcodes/indexes were pooled together as one multiplexing library to obtain 1 to 2 G next-generation sequencing (NGS) raw data. The sequencing reads were analyzed using an in-house script (refer to Methods for details), and the T-DNA integration sites (TISs) were validated by PCR and Sanger sequencing. Refer to Supplementary Table S1 for oligos used for Tn5 assembly and PCR.

The workflow of TTLOC is summarized in Fig. 1, including Tn5 assembly, tagmentation, PCR amplification, library pooling and NGS, data analysis, and PCR validation. TTLOC uses a hyperactive Tn5 transposase assembled with customized Illumina-compatible adapter mix (ME/ME-rev:ME-A/ME-rev =1:1, 50 µM, Supplementary Table S1), in which ME and ME-rev are the 19 bp Tn5 ME transposon sequence and its reverse & complementary sequence, respectively, while ME-A is ME sequence with a 14 bp 5′ bridge sequence compatible with P5 barcoding primer (N50X) for Illumina paired-end sequencing. Thus, the customized adapter mix has P5 bridge sequence but without P7 bridge sequence. Accordingly, the assembled Tn5 transposase did tagmentation of the genomic DNA of transgenic plants, resulting in DNA products with a P5 bridge sequence at one end but without P7 bridge sequences at the other end (Fig. 1). Subsequently, two separate PCR amplifications were conducted to retrieve the left TIS and right TIS, respectively. In each PCR, there are three primers, two of which are the shared P5 and P7 barcoding primers while the other one is the region-specific bridge primer for either LB or RB that has the P7 bridge sequences at its 5′ end, resulting as the amplification of the DNA fragments containing T-DNA and their flanking genomic sequences (Fig. 1, Supplementary Fig. S1, Supplementary Table S1). An example of indexing strategy for one pooled library was indicated, for each six independent samples, use the same P5 barcoding primer (e.g. N501) for all six samples and different P7 barcoding primer (e.g. N701 to N706, respectively for each sample; Fig. 1, refer to Methods part for details). PCR products of each six samples with barcode identifiers were pooled together as a multiplexing library for paired-end Illumina sequencing to obtain 1 to 2 G of raw data. NGS reads were then subjected to bioinformatic analysis (refer to Methods for details) using an in-house script (https://github.com/ShouliFeng2020/TTLOC) and further validation by PCR and Sanger sequencing (Fig. 1).

**Figure 2.**
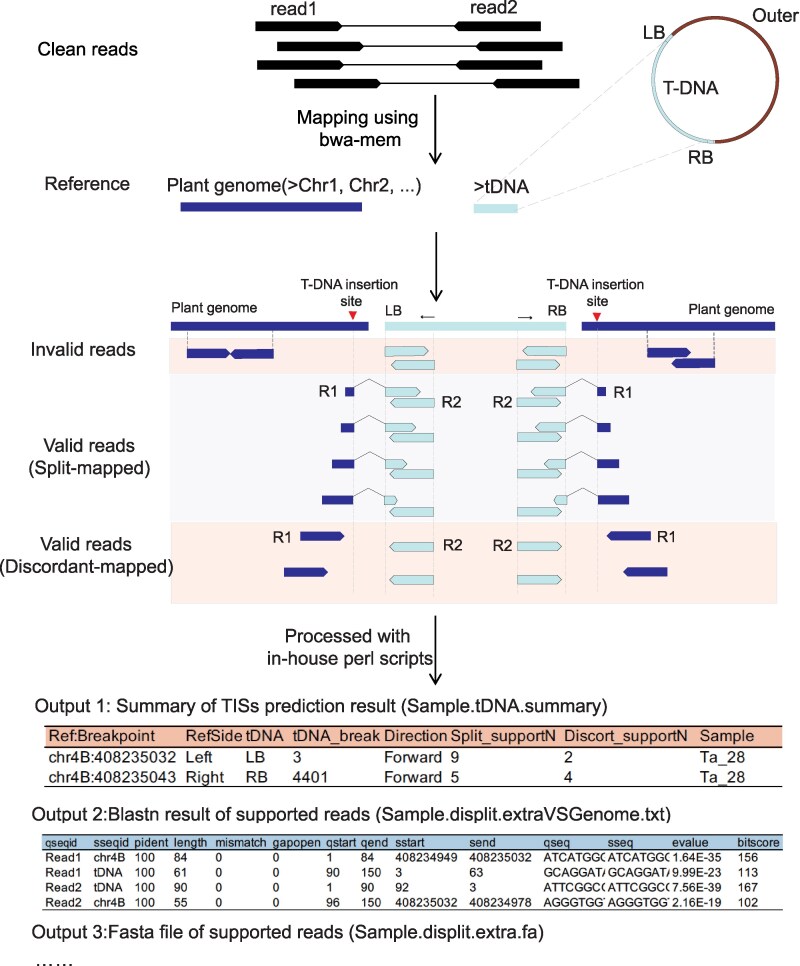
Procedure for bioinformatic analysis of TTLOC dataset. The next-generation sequencing (NGS) data after quality control was subjected to bwa software for alignment against reference sequences (merged file of T-DNA and plant genome sequences). According to the mapping results, invalid reads only mapped to plant genome or T-DNA; the valid reads include split-mapped and discordant-mapped reads supported the TISs. Split-mapped reads: sequence-through reads that span across the junction between the plant genome and the integrated T-DNA; Discordant-mapped reads: paired-end reads that connect the plant genome and the integrated T-DNA but do not sequence-through. The alignment results were processed with an in-house script available at https://github.com/ShouliFeng2020/TTLOC, resulting in output file 1 with summary information including sample name (“Sample”), break side of the genome (“Ref:Breakpoint”), insertion type (“RefSide”, “tDNA” and “Direction”), number of supported split- and discordant- reads (“Split_supportN”, “Discort_supportN”); output file 2 with blastn results of supported reads, and output file 3 with sequences of supported reads in fasta format. All the predicted TISs can be manually checked referring to the 3 major output files.

TTLOC generates three types of reads (Fig. 2): (1) invalid read pairs, which solely map to either the plant genome or the T-DNA sequences and offer no valuable information regarding the TISs; (2) valid read pairs with split-mapping, they are sequence-through reads that span across the junction connecting the plant genome and the integrated T-DNA, thereby precisely pinpointing the TISs; (3) valid read pairs with discordant mapping. Although they connect the plant genome and the integrated T-DNA, they do not sequence-through due to the limited sequencing length of NGS PE150 platform. Nevertheless, they furnish supplementary evidence to corroborate the TISs. A workflow for NGS data analysis is presented (Supplementary Fig. S2A); generated output files mainly include: (1) a summary output file entitled “sample.tDNA.summary” with the information of predicted TISs including sample name (“Sample”), insertion sites (“Ref:Breakpoint”), insertion type (“RefSide”, “tDNA” and “Direction”), number of supported split-mapped reads (“Split_supportN”) and discordantly-mapped reads (“Discort_supportN”) (Fig. 2); (2) a fasta file with detailed sequences of all supported reads; (3) BLAST results of all supported reads; and (4) 2,000 bp flanking genomic sequences of the predicted TISs, which supports primer design for the further PCR confirmation.

### TTLOC efficiently recovers TISs from transgenic Arabidopsis plants

**Figure 3.**
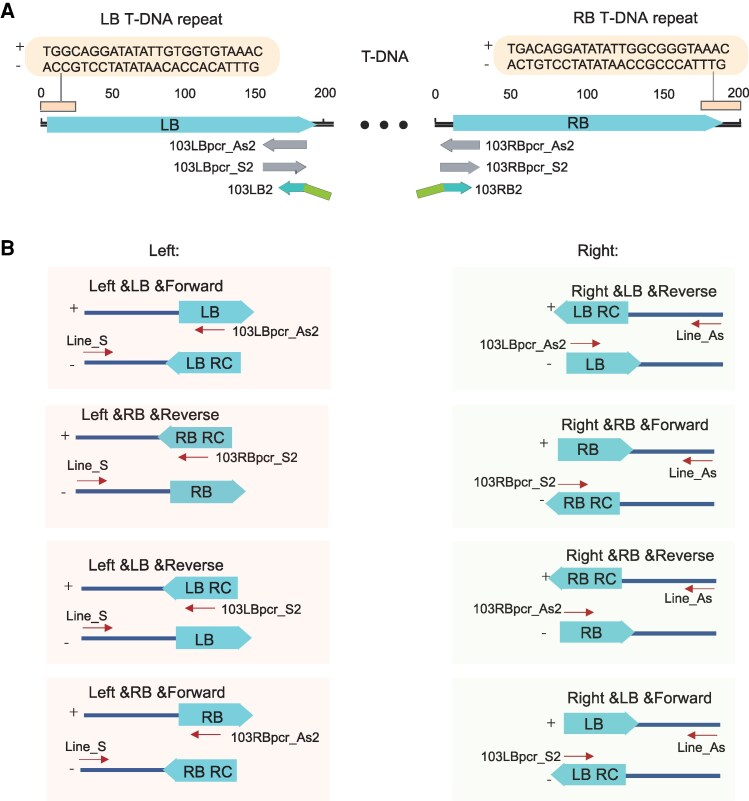
Primer design strategies for TIS confirmation by regular PCR. **A)** Schematic diagram showed the sequences of left border (LB) and right border (RB) repeat of T-DNA, we defined the upper strand indicated as the forward strand (+). The bridge adaptors (e.g. 103LB2, 103RB2 for T-DNA backbone pEarleyGate 103) for TTLOC library construction and its related common primers (e.g. 103LBpcr_As2, 103LBpcr_S2, 103RBpcr_As2, 103RBpcr_S2) for PCR confirmation were indicated. **B)** Schematic diagram showed the arrangement of different integration patterns with information of break side (“RefSide”), type of T-DNA (“tDNA”), and its direction (“Direction”). The primer pairs for PCR validation were recommended. As indicated, one site-specific primer combined with one common primer to LB or RB was used in each reaction. Line_S: upstream sense primer specific to flanking genome sequences of each TIS, Line_As: downstream antisense primer. Refer to Supplementary Table S1 for detailed sequences of primers used for PCR validation. Refer to Supplementary Table S2 for the primer pairs used for PCR validation of each T-DNA integration sites (TISs) in this study.

We first generated transgenic Arabidopsis (*A. thaliana,* Col-0) plants by expressing the exogenous *DsRed2* gene (Nishizawa et al. 2006) using Agrobacterium-mediated floral dip transformation. Then, TTLOC approach was applied to identify TISs in T1 transgenic Arabidopsis plants. A full TIS is composed of 2 insertion types: split reads supported the left and right TISs with the coordinates of the plant genome and the binary vector as well as mapping direction of T-DNA insertion type (“RefSide”, “tDNA” and “Direction” information in “sample.tDNA.summary” output file). In this study, we defined the upper strand between LB and RB repeat sequences as forward strand (+) (Fig. 3A). If the left and right TIS were considered separately, 8 possible insertion types were illustrated in a diagram (Fig. 3B), with a strategy for design of PCR validation primers shown, for each insertion type, a shared primer specific to the LB or RB region, and another upstream or downstream (∼500 bp) primer specific to flanking genome were recommended as primer pairs in PCR amplifications (Fig. 3B). The PCR products were Sanger-sequenced and the TISs were confirmed by blastn against the Arabidopsis genome sequences (Supplementary Fig. S2B).

**Figure 4.**
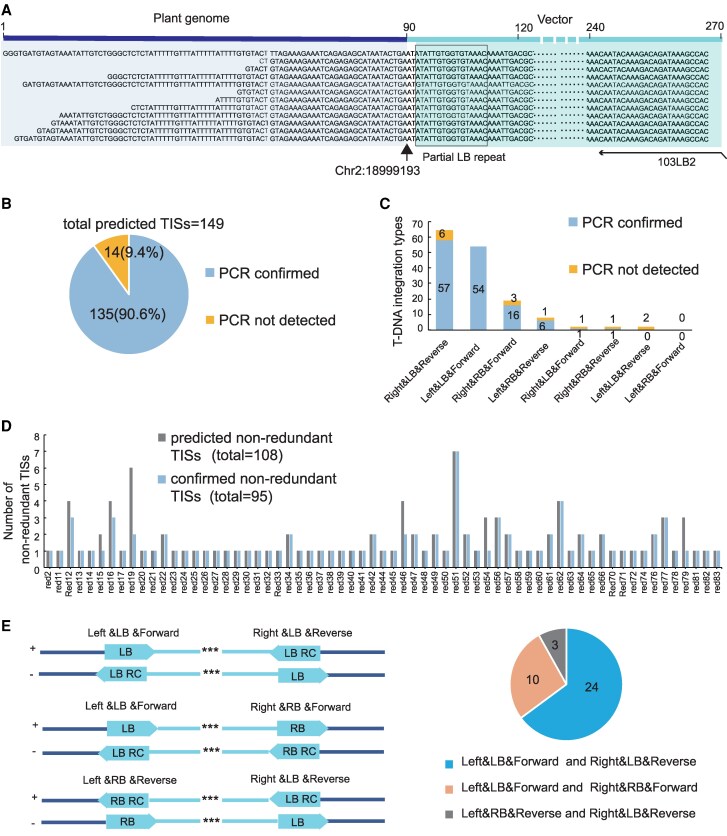
TTLOC efficiently recovered TISs in transgenic Arabidopsis plants. **A)** Representative multiple non-redundant split-mapped reads supported the integration site of left border (LB) region from T-DNA. The sequences of the genome part (marked in purple background) and the LB region (marked in blue background) were indicated. **B)** Graphic summary of recovered TISs by TTLOC from 65 transgenic Arabidopsis lines. Number of totally predicted and PCR-validated T-DNA integration sites (TISs) were indicated. **C)** Summary of insertion patterns for the total predicted 149 TISs. **D)** Number of non-redundant TISs in each line. Number of predicted TISs and PCR-validated ones were indicated. **E)** A graphical overview showed organization of 3 major full T-DNA insertion pattern recovered from transgenic Arabidopsis plants and their occurrence.

TTLOC recovered TISs from 65 of 74 independent transgenic lines. In total, we recovered 149 TISs that supported 108 non-redundant insertions from 65 independent Arabidopsis lines, of which 90.6% (135 TISs supported 95 non-redundant insertions) were successfully confirmed by PCR and Sanger sequencing (Fig. 4, A to D, Supplementary Table S2). We summarize the different types of TISs, in which the “Right&LB&Reverse” and “Left&LB&Forward” patterns accounted for 85.4% of the total PCR-validated TISs (Fig. 4C). The number of recovered non-redundant TISs in each independent Arabidopsis line was summarized, with single or multiple recovered TISs up to 7 (Fig. 4D). Thirty-seven out of 95 (∼39%) non-redundant TISs were recovered as full T-DNAs (i.e. with information of both left and right insertion border), in which 24 out of 37 (∼65%) TISs were a combination of “Left&LB&Forward” and “Right&LB&Reverse” (Fig. 4E).

**Figure 5.**
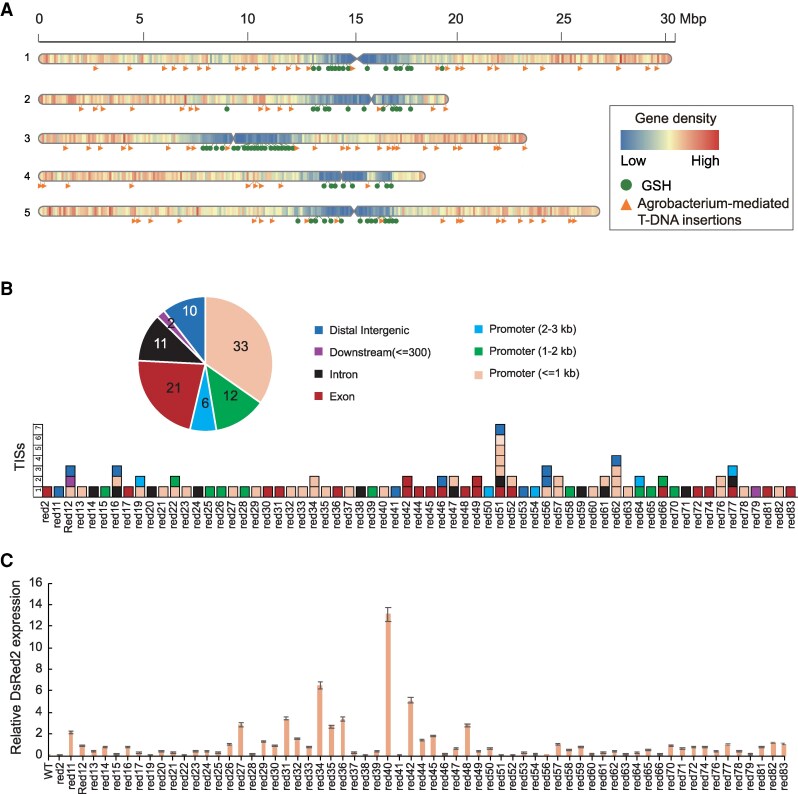
Analysis of TISs and expression of exogenous *DsRed*2 gene. **A)** A graphical overview of insertion events mediated by Agrobacterium transformation (*n* = 95) and the candidate genomic safe harbors (GSHs) in Arabidopsis plants. The *x* axis represents chromosome size; the *y* axis represents chromosome number; orange triangles represent Agrobacterium-mediated insertions; green circles represent candidate GSHs according to the criteria for GSHs prediction (Aznauryan et al. 2022; Sun et al. 2024); and the color in the chromosome indicates density of coding genes. Blue: low gene density; Red: high gene density. **B)** Graphic summary of gene-based annotation of total 95 T-DNA integration sites (TISs) and the summary of TISs in each transgenic Arabidopsis line. **C)** Relative expression of *Dsred2* mRNA by RT-qPCR. Assay was performed in triplicates for each sample. Data were presented as mean ± standard deviation (mean ± SD).

We further performed the annotation of validated TISs in 65 transgenic Arabidopsis lines and found that TISs are randomly distributed in each chromosome and are less likely inserted into the pericentromeric region, which has the lower gene density (Fig. 5A). We also predicted genome safer harbors (GSHs) using the criteria previously described (Aznauryan et al. 2022; Sun et al. 2024). We identified 70 candidate GSH regions in the Arabidopsis genome spanning a total of 602 kb (Supplementary Table S3), most of which were distributed in pericentromeric region (Fig. 5A), indicating that the TISs are unlikely overlapped with the predicted GSHs. We also conducted gene-level annotation of TISs, which indicated that 33 (34.7%), 12 (12.6%), 6 (6.3%), 11 (11.6%), 21 (22.1%), and 2 (2.1%) TISs were inserted at promoter (≤1 kb), promoter (1 to 2 kb), promoter (2 to 3 kb), intron, exon, downstream (≤300 bp) of the nearest gene, respectively. Moreover, 10 (10.5%) TISs were distal intergenic (>3 kb) inserted (Fig. 5B), resulting in varied expression levels of the exogenous *DsRed2* gene (Fig. 5C).

### TTLOC recovery of TISs is genome independent and applicable to large and complex plant genomes

**Figure 6.**
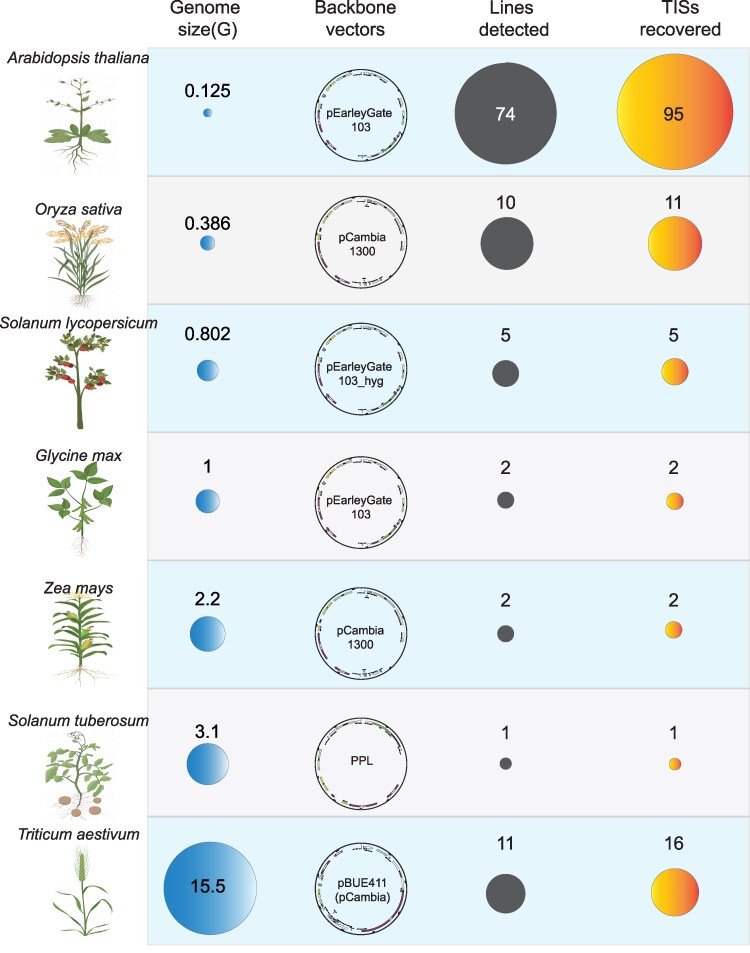
Summary of non-redundant T-DNA integration sites (TISs) recovered from multiple plant genomes using TTLOC. Refer to Supplementary Table S1 for detailed primer sequences for library construction and PCR confirmation. Refer to Supplementary Table S2 for information of TISs in each sample and the PCR validation results.

We further verified the feasibility of TTLOC in complex polyploid crop genomes after the successful application of TTLOC in the model plant of Arabidopsis, which has a much smaller genome compared with many plant genomes (The Arabidopsis Genome Initiative 2000; The Potato Genome Sequencing Consortium 2011; Walkowiak et al. 2020). We applied TTLOC to recover more T-DNA-transformed plants, including independent transgenic lines of monocots: wheat (*T. aestivum*, 11 lines), rice (*O. sativa*, 10 lines), and maize (*Zea mays*, 2 lines), as well as eudicots: tomato (*S. lycopersicum*, 5 lines), soybean (*G. max*, 2 lines), and potato (*S. tuberosum*, 1 line). These transgenic plants have multiple T-DNA backbone vectors, mainly over-expression vectors based on the “pEarleyGate” (Earley et al. 2006) and “pCambia” (Hajdukiewicz et al. 1994) vectors in plants and pCambia backbone-derived genome editing vector pBUE411 (Xing et al. 2014;  Fig. 6). Accordingly, the bridge primers with LB or RB region specific sequences for each backbone vector were designed (Supplementary Table S1). We determined the TISs in multiple genomes according to the same TTLOC approach as in Arabidopsis and successfully recovered 21 non-redundant TISs in multiple plants of rice, tomato, soybean, maize, and potato (Fig. 6, Supplementary Table S2), covering 92% of the detected plant lines.

**Figure 7.**
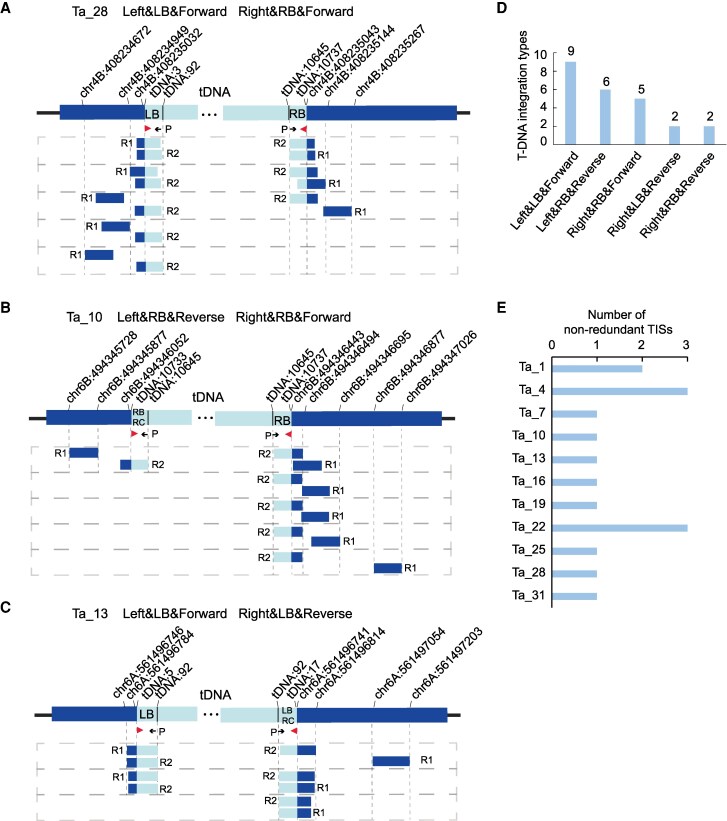
TTLOC efficiently recovered T-DNA integration sites (TISs) in transgenic wheat plants. **A–C)** Representative recovered TISs from transgenic wheat showed multiple insertion pattern. Non-redundant paired reads supporting the insertion patterns were indicated. R1: Read 1 started with genome sequences; R2: Read 2 started with T-DNA left border (LB) or right border (RB) sequences. The red triangle indicated the position and direction of partial LB or RB repeat sequences after T-DNA integration. The black arrow indicated the position and direction of bridge adaptor primer (P) in TTLOC library construction. **A)** TIS (chr4B: 408235032) recovered from Ta_28 line with insertion pattern of “Left&LB&Forward” and “Right&RB&Forward”. **B)** TIS (chr6B: 494346052) recovered from Ta_10 with insertion pattern of “Left&RB&Reverse” and “Right&RB&Forward”. **C)** TIS (chr6A:561496741) recovered from Ta_13 with insertion pattern of “Left&RB&Reverse” and “Right&RB&Forward”. **D and E)** Graphic summary of insertion patterns for the total recovered TISs **D)** and the number of non-redundant TISs in each line **E)**.

We used hexaploid bread wheat (IWGSC 2018; 15.5 Gb genome compared with 0.386 Gb rice genome and 0.125 Gb Arabidopsis genome) to test the efficiency of the TTLOC approach on complex & large genomes (Fig. 6). We successfully recovered 16 non-redundant TISs from 11 independent transgenic wheat lines (Fig. 6), covering 100% of the tested lines (Supplementary Table S2). We showed the recovered TISs from three representative transgenic lines with supported split or discordant reads (Fig. 7, A to C), in which the lines Ta_28 has the ideal T-DNA insertion pattern between LB and RB repeat sequences (Left&LB&Forward; Right&RB&Forward; Fig. 7A), while the line Ta_10 has the T-DNA insertion pattern between 2 RBs (Left&RB&Reverse; Right&RB&Forward; Fig. 7B), and the line Ta_13 has the T-DNA insertion pattern between two LBs (Left&&LB&Forward; Right&LB&Reverse; Fig. 7C). The analysis of TTLOC data for complex and large polyploid plant genomes may be challenging as the genomes with high sequence similarity between subgenomes and abundance of repetitive elements (Walkowiak et al. 2020). For example, we have identified the supported split and discordant reads that were 100% mapped to 4 different genome sites for line Ta_13 (chr6A:561496741, chr7B:510767368, chr1B:308385483, chr4B:623836685), with 94% sequence identity at the flanking 740 bp region of the insertion site (Supplementary Fig. S3). We then designed PCR primers specific to each genome site to confirm that the TISs of Ta_13 is at chr6A (Fig. 7C, Supplementary Fig. S3, Supplementary Table S1). Summary of the insertion pattern of T-DNA LB or RB region demonstrated that “Left&LB&Forward”, “Left&RB&Reverse” and “Right&RB&Forward” account for 83.3% of the insertion pattern (Fig. 7D), and 1 to 3 non-redundant TISs are successfully recovered for each wheat line (Fig. 7E, Supplementary Table S2). Thus, the TTLOC approach combined with PCR-Sanger sequencing confirmation can recover TISs efficiently from complex genomes of transgenic plants.

### Comparison of the general characters of TAIL-PCR, WGS-based T-LOC and TTLOC

Finally, we conducted a comprehensive comparison for traditional TAIL-PCR, WGS-based T-LOC and TTLOC (Table 1). In terms of experimental procedure, time, and operation, the traditional TAIL-PCR requires 7 to 8 h for 3 rounds of specialized PCR and product purification for Sanger sequencing. While WGS-based T-LOC takes advantage of the NGS for TISs identification, with a straightforward procedure including library construction, NGS, and data analysis. The whole process takes 4 to 5 h for library construction and varied time for data processing depend on the size of plant genomes. In comparison, our TTLOC method has only 4 concise steps: tagmentation of genomic DNA by Tn5 (15 min), 1 round of PCR (1.5 h), product purification and library pooling (30 min), which only takes 2.5 h for library construction. Considering the molecular characters of T-DNA results, TAIL-PCR and TTLOC approaches resulted as copies, integration sites, flanking sequence of T-DNA, while the WGS-based T-LOC approach can predict more detailed information including the complete integrated sequences of T-DNA, detailed sequence changes (repetitions, deletions, inversions) of the flanking genomic sequences. The predicted TISs from both WGS-based T-LOC and TTLOC can be confirmed by PCR and Sanger sequencing.

**Table 1.** Comparison of the 3 different approaches for TIS analysis

Method	TAIL-PCR	WGS-based T-LOC	TTLOC
Primer design	3 special primers; 1 arbitrary degenerate primer	PCR confirmation primers	2 bridge primers; PCR confirmation primers
Procedure	3 rounds of specialized PCR, purification, Sanger sequencing, sequence analysis	DNA fragment, end repair, adaptor ligation, PCR, purification, pooling and NGS; data analysis	Tn5 Tagmentation, PCR, purification, pooling and NGS, data analysis
Time of assay	7 to 8 h to PCR products	4 to 5 h to NGS library	2.5 h to NGS library
Sequencing depth	N/A	30× depth	1 to 2 G
Data processing	Blastn	T-LOC pipeline	TTLOC pipeline
Throughput	Low	High	High
Molecular characters of T-DNA	copies, integration sites, flanking sequence	copies, integration sites, detailed sequence changes of flanking regions, full sequence of T-DNA	copies, integration sites, flanking sequence
Cost per sample	low ($5)	High ($51 to 1,184) depending on genome size	Low ($9 or 14), genome size independent

The cost of TAIL-PCR is mainly for the primers, DNA polymerase enzymes, and Sanger sequencing, which has an estimated cost of $5 per sample. WGS-based T-LOC costs $51 per sample for rice with 0.386 Gb in genome size to obtain the required > 30× sequencing depth for effective TISs analysis (Li et al. 2022; Sun et al. 2024), and the cost increased depends on the genome size of plants. For example, the cost increases by 23.2-fold ($1,184) for a large crop genome such as 15.5 Gb hexaploid wheat genome (IWGSC 2018). In contrast, the cost of TTLOC is $9/sample if using home-made Tn5 transposase and $14/sample when using commercially available Tn5 (Supplementary Table S4). For each pooled TTLOC library, 1 to 2 Gb of raw data were adequate for effective TISs prediction. Thus, the cost of the TTLOC is comparable to TAIL-PCR, but it can recover TISs in high-throughput like WGS-based T-LOC did. In summary, TTLOC is a concise, high-throughput and cost-effective approach for TIS recovery for transgenic plants.

## Discussion

Our data established TTLOC as a concise, powerful, and low-cost approach for TISs identification, especially benefiting crop with large genomes. According to the working principle (Fig. 1), TTLOC is theoretically applicable to any species with known information of T-DNA backbone and reference genome. We have tested the feasibility of TTLOC in genomes of Arabidopsis, rice, tomato, soybean, maize, potato, and wheat transformed with multiple commonly used vector backbones (Fig. 6). Our results showed that when we use the same transgenic backbone vector pEarleyGate 103 in Arabidopsis, soybean, and tomato, the same bridge primer can efficiently recover the TISs for different genomes (Fig. 6, Supplementary Table S2), indicating TTLOC recovery of TISs is genome independent. Hexaploid bread wheat has a large genome (15.5 Gb), the high sequence similarity between subgenomes and abundance of repetitive elements (about 85% of the genome) hampered TIS recovery. Our TTLOC approach successfully recovered 16 non-redundant TISs from 11 independent transgenic wheat lines, covering 100% of the tested lines (Fig. 6, Supplementary Table S2), indicating TTLOC is applicable to large and complex plant genomes. TTLOC requires NGS, but it constructs a NGS library based on known information of the T-DNA backbone that is genome size-independent. According to the known sequences of LB and RB regions in the T-DNA backbones used in plant transformation (Hajdukiewicz et al. 1994; Earley et al. 2006; Xing et al. 2014), shared bridge primers can be applied in TTLOC. However, bridge primer specific to LB or RB region may have non-specific amplifications in different genome, further assays are required to test more vector backbone in different species to obtain more optimized bridge primers for TTLOC. Moreover, an ideal TIS prediction consists of both left and right flanking information; however, in some cases, the truncated LB or RB regions which lacking the PCR bridge primer sequences lead to the missing of TTLOC prediction, and resulted as only the left or right half of the TIS information (Supplementary Table S2). Manual prediction and PCR confirmation based on the known half TIS is an alternative method; otherwise, re-design the bridge primer for another TTLOC PCR is recommended.

Since the development of plant transformation using Ti plasmid-derived vector (Herrera-Estrella et al. 1983; Herrera-Estrella et al. 1984), Agrobacterium-mediated stable transformation has been widely applied to create transgenic or genome editing plants for crop breeding. As a result, T-DNA insertion can cause genomic changes and affect the expression of neighbor coding or non-coding genes at TISs, which may affect the yield and nutrition of crops. Besides, the TISs with different chromatin epigenetic status usually affects the expression of T-DNA transferred exogenous genes. Also, multi-copies of TISs usually lead to post-transcriptional gene silencing (Tang et al. 2007). Considering these issues caused by random insertion of T-DNA, TISs recovery is of great importance for safety assessment of transgenic plants. Using a modified high-efficiency thermal asymmetric interlaced PCR (mhi-TAIL-PCR), the flanking sequences of TISs of transgenic rice lines from the genetic engineering “Purple Endosperm Rice” (Zhu et al. 2017) and “Astaxanthin Rice” (Zhu et al. 2018) were efficiently recovered. Using WGS-based T-LOC, 75 full TISs was evaluated from 48 transgenic rice that provide real and unbiased resources of T-DNA integration patterns (Li et al. 2022). In this study, TTLOC recovered 122 non-redundant TISs from multiple plant genomes including Arabidopsis, rice, soybean, tomato, potato, and wheat. All these 3 approaches can recover TISs efficiently with different characters as described in Table 1, we would suggest chose the appropriate method according to the purpose of the assay. When large-scale number of transgenic samples need to be verified, it is better to use TTLOC for high-throughput screening and then WGS-based T-LOC for further genome-wide confirmation for selected lines.

Genome engineering for plant synthetic biology and plant breeding have raised an urgent demand for the precise and efficient manipulation of large segments of DNA (Klompe et al. 2019; Sun et al. 2024; Zhang et al. 2025). As one of the 7 science technologies to watch in 2024 according to *Nature*, precise integration of large DNA sequences in genomes offers hope for endowing food crops with disease resistance and improved nutrition (Eisenstein 2024). Genomic safe harbors (GSHs) sites are characterized as those positions within the genome that can accommodate the integration of new genetic materials in such a way that guarantees the newly inserted genetic elements that can function in a predictable manner without alterations of the host genome that pose risks to the host cell or organism (Papapetrou and Schambach 2016). Plant GSHs site-specific integration of exogenous DNA allows the efficient and stable expression of genes that are of great significance for the creation of transgenic-free plants for crop genetic improvement. In theory, GSH sites should be located 5 kb away from protein-coding genes to avoid disrupting functional regions associated with gene expression (Sun et al. 2024). However, our data on the distribution of TISs in the Arabidopsis genome revealed that ∼90% of TISs were inserted within 2 kb near protein-coding genes (Fig. 5B). These TISs did not overlap with the predicted GSHs in Arabidopsis, which is consistent with the results in rice (Sun et al. 2024). Such contradictions pose more challenges for the selection of GSHs for site-specific integration of large segment of DNA, requiring new solutions for GSHs selection for crops. We found the insertion sites and copies of T-DNA lead to varied expression levels of the gene (Fig. 5, B and C), and multiple transgenic lines with relative higher expression of gene had TISs inserted into Arabidopsis genome sites near protein-coding genes (<2 kb). Thus, TTLOC approach might provide an alternative way for GSH selection: T-DNA-mediated transformation generate abundant random insertion sites as GSHs candidates, then the application of TTLOC recovered TISs in high-throughput, the following analysis of location characteristics, gene expression and plant phenotype can provide direct experimental evidence for GSH sites selection. Therefore, TTLOC is a promising strategy, which benefits basic plant science research, crop breeding for sustainable increase of yield and quality, and also benefit for selection of GSHs for trait stacking based plant synthetic biology.

## Materials and methods

### Assembly of Tn5 transposase

Assembly of Tn5 transposase was performed as described (Tao et al. 2023a, 2023b) except that the adaptor mix assembled with Tn5 transposase was different. Sequences of primers for generating adaptors are provided (Supplementary Table S1). ME-A, ME, and ME-rev was diluted in annealing buffer (10 mm Tris pH 8.0, 50 mm NaCl, 1 mm EDTA) to make a concentration of 100 *μ*M. In 2 PCR tubes, set up the following reactions by mixing 10 *μ*L ME with 10 *μ*L ME-rev (tube 1 for adaptor ME/ME-rev), and 10 *μ*L ME-A with 10 *μ*L ME-rev (tube 1 for adaptor ME-A/ME-rev); The adaptors were generated using the program in the PCR machine (heat lid, 75 °C for 15 min, 60 °C for 10 min, 50 °C for 10 min, 40 °C for 10 min, 25 °C for 30 min). The adaptor ME/ME-rev and adaptor ME-A/ME-rev were mixed at 1:1 ratio, designated as “adaptor mix” (50 *μ*M). Five microliters of commercially available Tn5 (10 pmol/*μ*L, Novoprotein, Catalog no. M045) was complexed with 1.2 *μ*L adaptor mix buffer at 37 °C for 60 min to assemble the Tn5 transposase (final concentration ∼8 pmol/*μ*L).

### DNA isolation

Plant genomic DNA was isolated as previously described (Tao et al. 2020), briefly, 50 to 100 mg of the ground plant leaves were placed in a 1.5-mL centrifuge tube and 500 *μ*L of DNA extraction buffer added as previously reported (Paterson et al. 1993). The tube was placed in a 65 °C water bath for 30 min for lysis. Then, 600 *μ*L phenol:chloroform:isoamyl alcohol was added to each tube before shaking, and the tube was centrifuged for 10 min at 13,000 × *g* at 4 °C to collect the supernatant (∼500 *μ*L). Five hundred microliters of chloroform was added to each tube, and the extraction repeated to collect the supernatant (∼500 *μ*L). One milliliter of 100% ethanol and 50 *μ*L NaAc (3 m, pH5.2) was added to the supernatant and was put on ice for 10 min, DNA was collected by centrifugation for 10 min at 13,000 × *g* at 4 °C. The DNA was washed using 75% ethanol, air dried, and dissolved in 50 to 100 *μ*L ddH_2_O, adjusted to 200 to 400 ng/*μ*L in concentration for TTLOC analysis. Genomic DNA of wild-type plants was isolated for control sample in the TTLOC library construction.

### Criteria for TTLOC bridge primer design

Three primers were used for TTLOC library construction, including P5 primer (N50X), P7 primer (N70X), and a bridge primer. Sequences of 8 P5 primers and 12 P7 primers for generating adaptors are provided (Supplementary Table S1). The bridge primer consists of P7 bridge sequence at 5′ end, ME sequence at middle, and LB/RB region specific sequence at 3′ end (Supplementary Table S1). Each LB and RB bridge primer was designed according to the sequence of LB and RB region in T-DNA backbone (Supplementary Table S5), generally 150 to 200 bp away from T-DNA LB repeat and RB repeat (Fig. 3A, Supplementary Table S1).

### TTLOC library construction

A step-by-step protocol is available in the Supplementary Methods. TTLOC protocol was mainly the tagmentation, PCR amplification, purification, and pooling. For Tn5 tagmentation reaction, set up the following tagment reaction (20 *μ*L): 4 *μ*L 5× Tn5 buffer (50 *μ*L Tris +20 *μ*L MgCl_2_ + 925 *μ*LH_2_O), 11.1 *μ*L H_2_O, 3.2 *μ*L 50% PEG8000, 0.5 *μ*LTn5 (4 pmoL), 0.2 *μ*L ATP, for each sample, 1 *μ*L (200 ng) genomic DNA of transgenic plants was added. In parallel, genomic DNA of wild-type plants was set up as a control sample. The tagmentation reaction was performed at 55 °C for 15 min in the PCR machine, after that, 1 *μ*L1% SDS was added and keep the tube at 72 °C for 10 min to stop the reaction. For TTLOC library, set up the following PCR (25 *μ*L): 5 *μ*L tagmentation products from above reaction, 2 *μ*L N50X (10 *μ*M), 2 *μ*L N70X (10 *μ*M), 2 *μ*L LB/RB bridge primer (1 *μ*M), 12.5 *μ*L of 2× PCR mix with high-fidelity DNA polymerase enzyme (TransGen Biotech, Catalog no. AS231), add H_2_O to a final volume of 25 *μ*L. For each 6 samples, use the same P5 barcoding primer (N50X, e.g. N501 for all 6 samples) and different P7 barcoding primers (N70X, e.g. N701 to N706 for each sample, respectively) for library pooling. We provided 8 N50X and 12 N70X primers with different index sequences for more samples, refer to Supplementary Table S1 for the detailed sequences. The reaction was performed using the program in the PCR machine (heat lid, 72 °C for 10 min; 95 °C for 3 min; 95 °C for 30 s, 55 °C for 30 s, 72 °C for 30 s, repeat for 28 to 30 cycles; 72 °C for 5 min; 16 °C for 5 min). After reaction, PCR products are purified using 25 *μ*L (1/2 volume) SPRI-based DNA clean beads. Pooled PCR products together as a multiplex library for each 6 samples described above and subjected to NGS to obtain 2 G raw data for large wheat genome and 1 G raw data for other plant genomes.

### Data processing

In principle, NGS data of TTLOC libraries are compatible with previous reported software/pipeline such as TDNAscan (Sun et al. 2019) and T-LOC (Li et al. 2022). A TTLOC pipeline was provided in this study (https://github.com/ShouliFeng2020/TTLOC). Briefly, fastp was used to convert raw data to clean data with default parameters (Chen et al. 2018). Genome sequences of Arabidopsis, rice, soybean, corn, tomato, and potato are from Phytozome (*A. thaliana* TAIR10, *O. sativa* v7.0, *G. max* Wm82.a4.v1, *Zea mays* Zm-B73-REFERENCE-NAM-5.0.55, *S. lycopersicum* ITAG5.0, *S. tuberosum* v6.1), the wheat genome sequence download from https://shigen.nig.ac.jp/wheat/komugi/genome/download.jsp (Ouyang et al. 2007; Lamesch et al. 2012; Pham et al. 2020; Hufford et al. 2021; Sato et al. 2021; Zhou et al. 2022). The fasta files of plant genome sequence and the full T-DNA sequence (the region between LB and RB repeats with a sequence title of “tDNA”) were merged into a new reference genome fasta file. The clean data were aligned to the reference genome using the bwa-mem (https://anaconda.org/bioconda/bwa) with default parameters (Li 2013). The split reads and discordant reads were extracted using the samblaster (https://anaconda.org/bioconda/samblaster) with the following parameters”–addMateTags –maxSplitCount 2 -s split.sam -o samblaster.sam –minNonOverlap 20 -d discord.sam” (Faust and Hall 2014). Afterward, an in-house Perl script was employed to parse and extract information of valid reads from the resulting split.sam and discord.sam files (https://github.com/ShouliFeng2020/TTLOC) to generate output files.

### PCR confirmation of TTLOC results

Regular PCR primers were designed using Primer Premier software as Fig. 3B described, one primer is specific to plant genomic DNA sequences upstream or downstream of the T-DNA integration sites, another primer is specific to LB or RB region of T-DNA. The PCR products (generally 350 to 700 bp in size) were confirmed by Sanger sequencing, and the T-DNA integration sites were further confirmed by BLAST analysis using Sanger sequencing results as query and plant genome as reference.

### RT-qPCR

RT-qPCR was performed as described (Tao et al. 2023a, 2023b). The Arabidopsis housekeeping gene *ACTIN 2* (AT3G18780) was used as reference gene for data normalization in the data analysis (Lilay et al. 2021). RT-qPCR was performed in triplicates for each sample.

### Bioinformatic prediction of genomic safe harbors (GSHs) in Arabidopsis

GSHs in Arabidopsis genome were predicted according to the criteria as described (Aznauryan et al. 2022). Annotations of Arabidopsis genome and coding genes are from Phytozome (*A. thaliana* TAIR10; Lamesch et al. 2012). Annotation of tRNA regions were annotated by tRNAscan-SE with default parameters and miRNA regions were annotated using cmscan align the Rfam database (Chan and Lowe 2019; Kalvari et al. 2021). LncRNAs were annotated according to described pipeline (Feng et al. 2023) using data from Bioproject PRJEB32665 and PRJNA494179. The centromeric regions were annotated by quarTeT CentroMiner (Lin et al. 2023). Lastly, GSHs were predicted using the criteria including 10 kb away from centromeres, 20 kb away from lncRNAs, 10 kb away from gene-encoding regions, 30 kb away from miRNAs, 20 kb away from tRNAs.

### Accession numbers

The TTLOC NGS data generated from this article can be found in the NCBI (National Center for Biotechnology Information) Sequence Read Archive (SRA) database under accession number PRJNA1121050.

## Supplementary Material

kiaf102_Supplementary_Data

## Data Availability

In-house Perl script was developed for prediction of TISs from NGS data, available at https://github.com/ShouliFeng2020/TTLOC

## Supplementary data

The following materials are available in the online version of this article.

**Supplementary Figure S1.** Characteristics of the TTLOC library.

**Supplementary Figure S2.** Workflow/strategies for TTLOC data processing and PCR confirmation of predicted TISs.

**Supplementary Figure S3.** PCR confirmation of TIS in the transgenic wheat line Ta_13 with reads that have multiple alignments.

**Supplementary Table S1.** Oligos used in this study.

**Supplementary Table S2.** Summary of T-DNA integration sites in this study.

**Supplementary Table S3.** Predicted GSH regions in Arabidopsis.

**Supplementary Table S4.** A cost estimation for Tn5-mediated T-LOC, TAIL-PCR and WGS-based T-LOC.

**Supplementary Table S5.** Sequences of the LB and RB regions in the T-DNA backbones used in this study.

**Supplementary Methods.** A step-by-step TTLOC protocol.

## Dive Curated Terms

The following phenotypic, genotypic, and functional terms are of significance to the work described in this paper:

SDS Gramene: AT1G14750
SDS Araport: AT1G14750

ATP CHEBI: CHEBI:15422
