# Supplementary material for: TTLOC: A Tn5 transposase-based approach to localize T-DNA integration sites
Source: Plant Physiol. 2025 Mar 25;197(4):kiaf102. doi: 10.1093/plphys/kiaf102 (PMC11961865; doi:10.1093/plphys/kiaf102)
Supplement: kiaf102_Supplementary_Data [file kiaf102_supplementary_data.pdf]

TTLOC: A Tn5 transposase-based approach to localize T-DNA integration sites

Xiao-Yuan Tao†[1*], Shou-Li Feng†[1], Xin-Jia Li[1], Yan-Jun Li[1], Wei Wang[2], Matthew Gilliham[3], Zhong-Hua Chen[4*], Sheng-Chun Xu[1,5*]

1 Biotechnology Institute, Xianghu Laboratory, Hangzhou, 311231, China
2 National Key Laboratory of Crop Genetics & Germplasm Enhancement and Utilization, College of Agriculture, Nanjing Agricultural University/Zhongshan Biological Breeding Laboratory/Collaborative Innovation Center for Modern Crop Production (CIC-MCP), Nanjing, Jiangsu 210095, China
3 ARC Centre of Excellence Plants for Space, School of Agriculture, Food and Wine, Waite Research Precinct, University of Adelaide, Glen Osmond, SA 5064, Australia
4 School of Science, Western Sydney University, Penrith, NSW 2751, Australia
5 Institute of Digital Agriculture, Zhejiang Academy of Agricultural Sciences, Hangzhou, 310021, China

†These authors contributed equally to this work.
*Author for correspondence: Sheng-Chun Xu (xushengchun@xhlab.ac.cn); Zhong-Hua Chen (z.chen@westernsydney.edu.au); Xiao-Yuan Tao (taoxiaoyuan@xhlab.ac.cn).

Running title: Tn5-based T-DNA localization

One-sentence summary: TTLOC is a Tn5 transposase-based tool for evaluation of genetically engineered plants, which is a promising tool in crop breeding and plant synthetic biology.

The author responsible for distribution of materials integral to the findings presented in this article in accordance with the policy described in the Instructions for Authors (https://academic.oup.com/plphys/pages/General-Instructions) is Sheng-Chun Xu (xushengchun@xhlab.ac.cn).

SUPPLEMENTARY FIGURES

Supplementary Figure S1

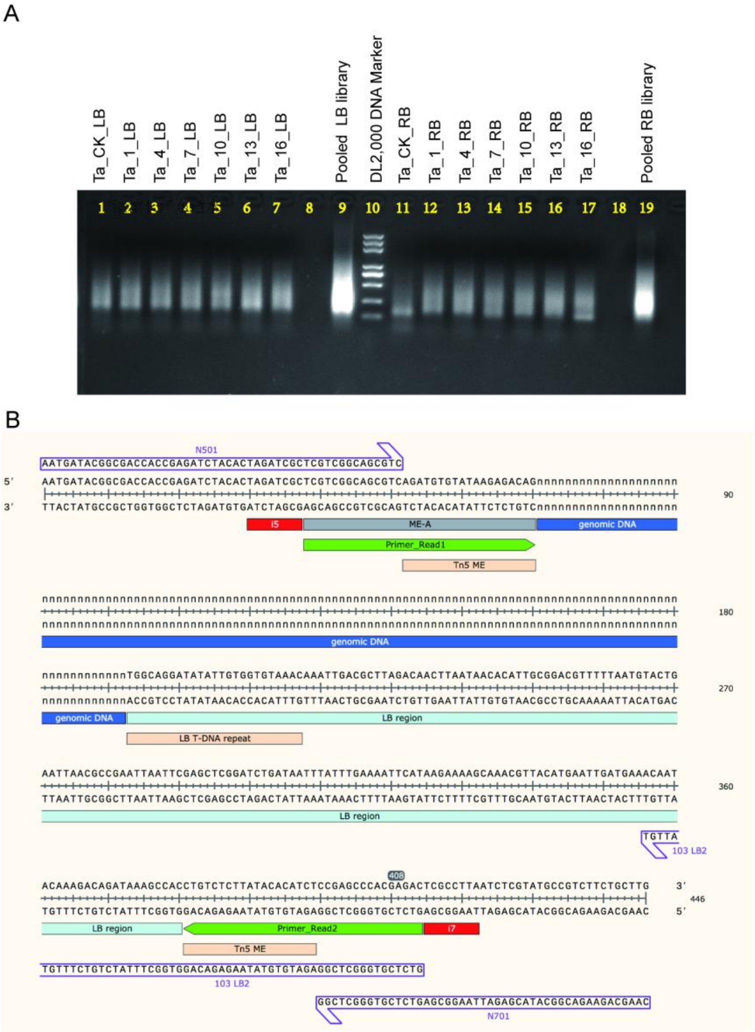

Supplementary Figure S1. Characteristics of TTLOC library. (**A**) Gel results of representative TTLOC samples. Gel electrophoresis detection was performed using 3 μL PCR products of each sample (lane 1-7, 11-17) and the pooled library after SPRI beads purification (lane 9 and 19). Pooled LB/RB library: mixture library of LB or RB PCR amplicons with different indexes. (**B**) Target library structure of TTLOC. The annotations of the sequences are indicated. Adaptor/primer sequences (N501, 103LB2, N701) are indicated by text box with an arrow. N501: a P5 barcoding primer; N701: a P7 barcoding primer; 103LB2: a bridge primer specific to LB region of pEarleyGate 103 vector, with the 15 bp 5' bridge sequence compatible with N701. Dark bule box: genomic DNA, n represents A, T, G, or C; Light blue box: LB region; Red box: 8 bp index 1 (i7) and index 2 (i5) sequences; Green box: sequencing primers for Read 1 and Read 2 in NGS ; Tn-ME: 19 bp Tn5 mosaic end (ME) transposon sequence; ME-A: adaptor sequences for Tn5 assembly; LB T-DNA repeat: T-DNA left border (LB) repeat, usually truncated after T-DNA insertion.

Supplementary Figure S2

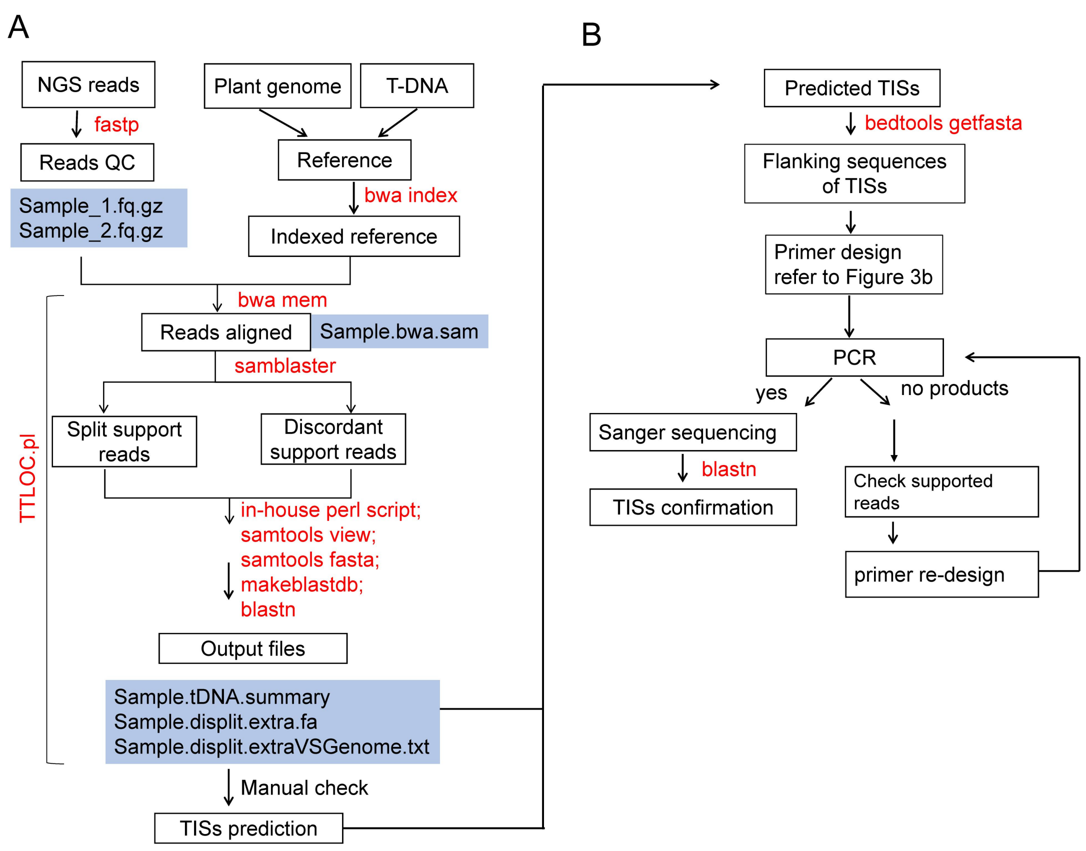

Supplementary Figure S2. Workflow/strategies for TTLOC data processing and PCR confirmation of predicted TISs. (A) TTLOC data processing workflow. An in-house script (TTLOC.pl) was available at https://github.com/ShouliFeng2020/TTLOC for TIS calling. The red words are software and codes used, and the text highlight in blue are resulting files. **(B)** Strategies for PCR confirmation of predicted TISs. Primer pairs were designed according to Figure 3b, including a primer specific to flanking genome sequences of each TIS and a common primer specific to LB or RB region. The PCR products are generally 350-700 bp in size and are confirmed by Sanger sequencing. The TISs were further confirmed by BLAST analysis using Sanger sequencing results as query and plant genome as reference.

Supplementary Figure S3

A

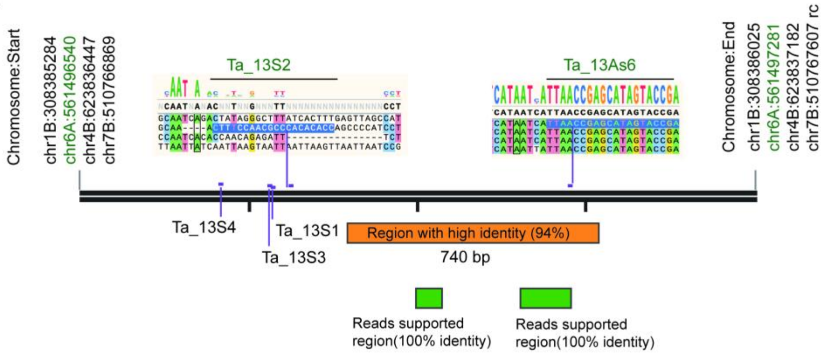

B

Top 4 blastn hits of PCR products (pBUELBpcr_As3&Ta_13S2):

qseqid	sseqid	pident	length	mismatch	gapope	qstart	qend	sstart	send	evalue	bitscore
TA-13S2-S-T_M13R_T	chr6A	100	363	0	0	128	490	561496721	561496359	0	671
TA-13S2-S-T_M13R_T	chr6A	93.96	149	6	2	342	490	219552179	219552324	1.30E-54	222
TA-13S2-S-T_M13R_T	chr6A	91.241	137	12	0	351	487	622554448	622554584	4.74E-44	187
TA-13S2-S-T_M13R_T	chr6A	91.241	137	12	0	351	487	622684855	622684991	4.74E-44	187

Top 4 blastn hits of PCR products (pBUELBpcr_As3&Ta_13As6):

qseqid	sseqid	pident	length	mismatch	gapope	qstart	qend	sstart	send	evalue	bitscore
TA-13AS6_PBUELBPCI	chr6A	100	455	0	0	16	470	561496741	561497195	0	841
TA-13AS6_PBUELBPCI	chr7B	99.121	455	4	0	16	470	510767411	510766957	0	819
TA-13AS6_PBUELBPCI	chr4B	97.582	455	9	1	16	470	623836642	623837094	0	778
TA-13AS6_PBUELBPCI	chr1B	96.937	457	10	2	16	470	308385483	308385937	0	763

Supplementary Figure S3. PCR confirmation of TISs in the transgenic wheat line Ta_13 with reads that have multiple alignments. (A) PCR primers designed for predicted TISs for line Ta_13. The supported split and discordant reads (indicated in green box) have multiple alignments: chr6A:561496741, chr7B:510767368, chr1B:308385483 and chr4B:623836685, these four predicted region had 94% sequence identity at the flanking 740 bp of the insertion site. Ta_13S1 to Ta_13S4: primers specific for upstream sequences of predicted TISs located on chr1B, chr6A, chr4B, and chr7B respectively. Ta_13As6: primer designed according to the homologous regions of four predicted TISs. Sequence alignments of predicted TIS regions were showed, Ta_13S2 and Ta_13As6 were highlighted in blue. (**B**) Blast results of PCR-Sanger sequencing data confirmed that the TIS of Ta_13 is at chr6A. The best hit was highlighted in green.

Supplementary Table 1. Oligos used in this study.

Primer name	Sequence (5'-3')	Usage
ME	AGATGTGTATAAGAGACAG	generating adaptors for Tn5 assembly
ME-rev	CTGTCTCTTATACACATCT(5'-Phosphate, 3'-AminolinkerC7)	generating adaptors for Tn5 assembly
ME-A	TCGTCGGCAGCGTCAGATGTGTATAAGAGACAG	generating adaptors for Tn5 assembly
N501	AATGATACGGCGACCACCGAGATCTACACTAGATCGCTCGTCGGCAGCGTC	NGS library P5 index primer
N502	AATGATACGGCGACCACCGAGATCTACACCTCTCTATTCGTCGGCAGCGTC	NGS library P5 index primer
N503	AATGATACGGCGACCACCGAGATCTACACTATCCTCTTCGTCGGCAGCGTC	NGS library P5 index primer
N504	AATGATACGGCGACCACCGAGATCTACACAGAGTAGATCGTCGGCAGCGTC	NGS library P5 index primer
N505	AATGATACGGCGACCACCGAGATCTACACGTAAGGAGTCGTCGGCAGCGTC	NGS library P5 index primer
N506	AATGATACGGCGACCACCGAGATCTACACACTGCATATCGTCGGCAGCGTC	NGS library P5 index primer
N507	AATGATACGGCGACCACCGAGATCTACACAAGGAGTATCGTCGGCAGCGTC	NGS library P5 index primer
N508	AATGATACGGCGACCACCGAGATCTACACCTAAGCCTTCGTCGGCAGCGTC	NGS library P5 index primer
N701	CAAGCAGAAGACGGCATACGAGATTAAGGCGAGTCTCGTGGGCTCGG	NGS library P7 index primer
N702	CAAGCAGAAGACGGCATACGAGATCGTACTAGGTCTCGTGGGCTCGG	NGS library P7 index primer
N703	CAAGCAGAAGACGGCATACGAGATAGGCAGAAGTCTCGTGGGCTCGG	NGS library P7 index primer
N704	CAAGCAGAAGACGGCATACGAGATTCCTGAGCGTCTCGTGGGCTCGG	NGS library P7 index primer
N705	CAAGCAGAAGACGGCATACGAGATGGACTCCTGTCTCGTGGGCTCGG	NGS library P7 index primer
N706	CAAGCAGAAGACGGCATACGAGATTAGGCATGGTCTCGTGGGCTCGG	NGS library P7 index primer
N707	CAAGCAGAAGACGGCATACGAGATCTCTCTACGTCTCGTGGGCTCGG	NGS library P7 index primer
N708	CAAGCAGAAGACGGCATACGAGATCAGAGAGGGTCTCGTGGGCTCGG	NGS library P7 index primer
N709	CAAGCAGAAGACGGCATACGAGATGCTACGCTGTCTCGTGGGCTCGG	NGS library P7 index primer
N710	CAAGCAGAAGACGGCATACGAGATCGAGGCTGGTCTCGTGGGCTCGG	NGS library P7 index primer
N711	CAAGCAGAAGACGGCATACGAGATAAGAGGCAGTCTCGTGGGCTCGG	NGS library P7 index primer
N712	CAAGCAGAAGACGGCATACGAGATGTAGAGGAGTCTCGTGGGCTCGG	NGS library P7 index primer
103LB2	GTCTCGTGGGCTCGGAGATGTGTATAAGAGACAGGTGGCTTTATCTGTCTTTGTATTGT	pEarleyGate 103 LB region specific primer with P7 bridge sequence, working as LB bridge primer
103RB2	GTCTCGTGGGCTCGGAGATGTGTATAAGAGACAGGCGTAATAGCGAAGAGGCCCGCA	pEarleyGate 103 RB region specific primer with P7 bridge sequence, working as RB bridge primer
pBUE_LB2	GTCTCGTGGGCTCGGAGATGTGTATAAGAGACAGAATCCAGTACTAAAATCCAGATCCC	pBUE411(pCambia) LB region specific primer with P7 bridge sequence, working as LB bridge primer
pBUE_RB2	GTCTCGTGGGCTCGGAGATGTGTATAAGAGACAGTTTCGCCAGCTGGCGTAATAGCGAA	pBUE411(pCambia) RB region specific primer with P7 bridge sequence, working as RB bridge primer
1300LB2	GTCTCGTGGGCTCGGAGATGTGTATAAGAGACAGaatccagtactaaaatccagatccc	pCambia1300 LB region specific primer with P7 bridge sequence, working as LB bridge primer
1300RB2	GTCTCGTGGGCTCGGAGATGTGTATAAGAGACAGtacccaacttaatcgccttgcagca	pCambia1300 RB region specific primer with P7 bridge sequence, working as RB bridge primer
1300LB3	GTCTCGTGGGCTCGGAGATGTGTATAAGAGACAGCCCCCGAATTAATTCGGCG	pCambia1300 LB region specific primer with P7 bridge sequence, working as LB bridge primer
1300RB3	GTCTCGTGGGCTCGGAGATGTGTATAAGAGACAGTGGCGAATGCTAGAGCAGC	pCambia1300 RB region specific primer with P7 bridge sequence, working as RB bridge primer
PPL_LB	GTCTCGTGGGCTCGGAGATGTGTATAAGAGACAGCTAGGATAAATTATCGCGCGCGGTG	PPL LB region specific primer with P7 bridge sequence, working as LB bridge primer
PPL_RB	GTCTCGTGGGCTCGGAGATGTGTATAAGAGACAGAGTCTCACCCAAGCTTGGG	PPL RB region specific primer with P7 bridge sequence, working as RB bridge primer
103LBpcr_S2	GATGAAACAATACAAAGACAGATAAAGCCAC	PCR confirmation primer specific to LB region, sense
103LBpcr_As2	GTGGCTTTATCTGTCTTTGTATTGTTTCATC	PCR confirmation primer specific to LB region, anti-sense

103RBpcr_S2	CCAACTTAATCGCCTTGCAGCACATC	PCR confirmation primer specific to RB region,sense
103RBpcr_As2	GATGTGCTGCAAGGCGATTAAGTTGG	PCR confirmation primer specific to RB region, anti-sense
pBUELBpcr_S2	GGGATCTGGATTTTAGTACTGGATT	PCR confirmation primer specific to LB region, sense
pBUELBpcr_S3	ACTTAATAACACATTGCGGACG	PCR confirmation primer specific to LB region, sense
pBUELBpcr_As2	AATCCAGTACTAAAATCCAGATCCC	PCR confirmation primer specific to LB region, anti-sense
pBUELBpcr_As3	CGTCCGCAATGTGTTATTAAGT	PCR confirmation primer specific to LB region, anti-sense
pBUERBpcr_S2	TTTCGCCAGCTGGCGTAATAGCGAA	PCR confirmation primer specific to RB region, sense
pBUERBpcr_S3	CCTGAATGGCGAATGCTAGA	PCR confirmation primer specific to RB region,sense
pBUERBpcr_As2	TTCGCTATTACGCCAGCTGGCGAAA	PCR confirmation primer specific to RB region,anti-sense
pBUERBpcr_As3	TCTAGCATTCGCCATTCAGG	PCR confirmation primer specific to RB region,anti-sense
1300LBpcr_S2	gggatctggattttagtactggatt	PCR confirmation primer specific to LB region, sense
1300LBpcr_As2	aatccagtactaaaatccagatccc	PCR confirmation primer specific to LB region, anti-sense
1300RBpcr_As2	tgctgcaaggcgattaagttgggta	PCR confirmation primer specific to RB region,sense
1300RBpcr_S2	tacccaacttaatcgccttgcagca	PCR confirmation primer specific to RB region, anti-sense
PPL_LBpcr_S2	CACCGCGCGCGATAATTTATCCTAG	PCR confirmation primer specific to LB region, sense
PPL_LBpcr_As2	CTAGGATAAATTATCGCGCGCGGTG	PCR confirmation primer specific to LB region, anti-sense
PPL_RBpcr_S2	CCGAGGAGGTTTCCCGATATTACCC	PCR confirmation primer specific to RB region, sense
PPL_RBpcr_As2	GGGTAATATCGGGAAACCTCCTCGG	PCR confirmation primer specific to RB region,anti-sense
PPL_RBpcr_As3	CCCAAGCTTGGGTGAGACT	PCR confirmation primer specific to RB region, anti-sense
BlpR_2As	CTACACCCACCTGCTGAAGTC	PCR confirmation primer specific to BlpR，anti-sense
Hyg_S	gcccaagctgcatcatcgaa	PCR confirmation primer specific to Hyg，sense
Hyg_As	ttcgatgatgcagcttgggc	PCR confirmation primer specific to Hyg，anti-sense
Ocster_S	CGCATGGGTGAGATTCCTTG	PCR confirmation primer specific to Ocs termiantor，sense
Ocster_As	CAAGGAATCTCACCCATGCG	PCR confirmation primer specific to Ocs termiantor，anti-sense
noster_S	gaatcctgttgccggtcttg	PCR confirmation primer specific to nos termiantor，sense
noster_As	caagaccggcaacaggattc	PCR confirmation primer specific to nos termiantor，anti-sense
Kan_S	AGCAATATCACGGGTAGCCA	PCR confirmation primer specific to Kan，sense
Kan_As	TGGCTACCCGTGATATTGCT	PCR confirmation primer specific to Kan，anti-sense
pTaU3_S	gccgccaactataacagcaa	PCR confirmation primer specific to TaU3 promoter，sense
pTaU3_As	ttgctgttatagttggcggc	PCR confirmation primer specific to TaU3 promoter，anti-sense
red11_As	GAGAGACGGATAATACGCTGCGTTTCGATG	Genome-specific PCR confirmation primer
red11_S	CGTTTCTGCGTGAGATGCAACTTGGCATG	Genome-specific PCR confirmation primer
red12_As	CGTCTCAGCTAACACTGCTGCGTTA	Genome-specific PCR confirmation primer
red12_As_i2	GATTCCGATCGGAACCCTGAAATCAGAG	Genome-specific PCR confirmation primer
red12_As_i3	CCGACTACAAGAGCGATTTACGACTC	Genome-specific PCR confirmation primer
red12_As_i4	CTTGCATGTTAAGGTCTCTCTACTCTAC	Genome-specific PCR confirmation primer
red12_S	CCGATCCGAGGAACGAAATTTTCACAAC	Genome-specific PCR confirmation primer
red12_S_i2	CTCGTTCTCTCTTCTTGTTCGTTGCTTA	Genome-specific PCR confirmation primer
red13_As	CTTCTTTAGCTTCTTCAAGACTAGGAGG	Genome-specific PCR confirmation primer
red13_S	GGATCTAAAATATTAGACCGCTCGCTAA	Genome-specific PCR confirmation primer
red14_As	CAAGAATTTGACACGTTGTTGAGCTAAATC	Genome-specific PCR confirmation primer
red14_S	GTTGTCAATAGTCAATGGCTCACAGTCAC	Genome-specific PCR confirmation primer
red15_As_1	GTGTTTGGCAAATATGATAGTAGAAGGTATC	Genome-specific PCR confirmation primer
red15_As_2	GAACAAAATAAGGTGTGTTTGAGTAATGG	Genome-specific PCR confirmation primer

red15_S_1	CATGGAAATTTGGGTTTGCCTGATCTAG	Genome-specific PCR confirmation primer
red15_S_2	CAAATGCAAACATGGAAATTTGGGTTTGCC	Genome-specific PCR confirmation primer
red15_S_i2	CGTGATGTTGTTTCTGGCTCATCATC	Genome-specific PCR confirmation primer
red16_As	TAGGAAGCTTGGAAGTGGATCATTCGGTG	Genome-specific PCR confirmation primer
red16_As_i2	CCAAAGACTCATATGGGCTTTGGCT	Genome-specific PCR confirmation primer
red16_As_i3	GGGTAGGAAGCTTGGAAGTGGATCAT	Genome-specific PCR confirmation primer
red16_As_i4	TCTTCTTATCATGATAGGCTTTGGTGC	Genome-specific PCR confirmation primer
red16_S	CACATCCATGAGACGGACGAGTAAG	Genome-specific PCR confirmation primer
red16_S_i2	CTCCCAACATATCAAATCAAATCACATAAAGG	Genome-specific PCR confirmation primer
red16_S_i3	CGACATAAATGCTGAATTAGTTGAGGACA	Genome-specific PCR confirmation primer
red16_S_i4	GAGTCGTCGATAAGCATCTGACGAGA	Genome-specific PCR confirmation primer
red17_As	GTGGGACTTAGCTATCTACAATTCTGC	Genome-specific PCR confirmation primer
red17_As_i2	CAATTCCACCATCAGTTGTTAGTCGT	Genome-specific PCR confirmation primer
red17_S	AGACATGAATCGGAGCCAAGATGCTC	Genome-specific PCR confirmation primer
red18_As	GCAGGTTGGGCAAATGCCCAGATTC	Genome-specific PCR confirmation primer
red18_As_i2	GAGACAGAAATATGACATAGCACACCC	Genome-specific PCR confirmation primer
red18_As_i3	GGATTTAGCATCCCCACATCTTCTC	Genome-specific PCR confirmation primer
red18_As_i4	AGGTTCTCACCATTTCCTTCCTTGTTTC	Genome-specific PCR confirmation primer
red18_As_i5	CTTTATCATGCACCGGAGGAGGAG	Genome-specific PCR confirmation primer
red18_S	GATTCATTTCTGATTCAAACCAATTTTCCCC	Genome-specific PCR confirmation primer
red18_S_i2	TGGAAAGATCTCCGCTTGCCCATA	Genome-specific PCR confirmation primer
red18_S_i3	GGTTGCTTAGAGACAAAGTTAGGGTTTTGC	Genome-specific PCR confirmation primer
red18_S_i4	CCCTTGTAAATCTTTCAAAACACTTTTAAACCC	Genome-specific PCR confirmation primer
red19_As	GCATTCCTGAAAATCATGCATGCGACGAAG	Genome-specific PCR confirmation primer
red19_As_i2	GAAATTGAACGCCGAAGAACAGAGGAAG	Genome-specific PCR confirmation primer
red19_As_i3	GCTCCATGTCAAAATGGGTTACCATAC	Genome-specific PCR confirmation primer
red19_S	CAAGTGCAAACAATGGGAGATATTGTTCC	Genome-specific PCR confirmation primer
red19_S_i2	GTTCTTCTGAACAGTCTCCGTGAAG	Genome-specific PCR confirmation primer
red2_As	CAGGGTCGTAGGAACTCTGTAAGAATC	Genome-specific PCR confirmation primer
red2_S	GTTTGCTTCACATAATGCCAAATCATCC	Genome-specific PCR confirmation primer
red20_As	GTATCGACCTATCAATCAAGTAGACAGTGG	Genome-specific PCR confirmation primer
red20_As_i2	GAGGAGTGTGTATATAATTGGAAACCTGG	Genome-specific PCR confirmation primer
red20_S	CTTCACCACAAGAAGGCTTCTATCTTAAG	Genome-specific PCR confirmation primer
red21_S	CGACGAGGAGTGTGGATTCTGTTG	Genome-specific PCR confirmation primer
red22_As_i2	ACTATTTAGGTCGGCAAAACGTTACATG	Genome-specific PCR confirmation primer
red22_As_i3	CATGAAACTGAGATAAAGAGGAGTGTGT	Genome-specific PCR confirmation primer
red22_S	TCTACACGCGCCTTCAATGTTTCAG	Genome-specific PCR confirmation primer
red22_S_i2	GAGTTGCTTGTTCATTACAGGAGGAAAG	Genome-specific PCR confirmation primer
red23_As	GCACTCTGGTGACGAGAACAAAGAC	Genome-specific PCR confirmation primer
red23_S	CGGAGCAACTTATTTTGACTGATCGTG	Genome-specific PCR confirmation primer
red24_As	GTTTGAGATTTGTGGTAATCTCGTCTACC	Genome-specific PCR confirmation primer
red25_As	GACCTTCACGACTTCACACGAAC	Genome-specific PCR confirmation primer
red25_S	GAAAACACAACGACGAGGACACTTCAA	Genome-specific PCR confirmation primer
red26_As	ACATTTCAGATGGATTTGACTAGCCC	Genome-specific PCR confirmation primer
red26_S	AGTTCACGCAAAGGTCACAACACT	Genome-specific PCR confirmation primer
red27_S	GGATCCCAGACTTTAATGGAAGGTCC	Genome-specific PCR confirmation primer
red28_As	CTACTCCATAATTTTGGGTGACTTACCAC	Genome-specific PCR confirmation primer
red29_As	CAACCTGATAGAACAAAGCGTTGG	Genome-specific PCR confirmation primer
red30_As	GCTCAGCAGGAGTTAGAAGTTTACC	Genome-specific PCR confirmation primer
red30_S	GACCCGAAGAGAGTAAATTAGTTAGAGCA	Genome-specific PCR confirmation primer
red31_As	GGCCCAAAGATAGACATAACAGTGTC	Genome-specific PCR confirmation primer
red32_As	CGGAGCATCTTTGGATAAGATGGTAAC	Genome-specific PCR confirmation primer
red32_S	CCTGAGAAGGAGTAGTTGAGAGAGCC	Genome-specific PCR confirmation primer
red33_As	GGTAGAATCTGGTGTCTGTGGGAGAAG	Genome-specific PCR confirmation primer
red34_As	TCATGTTCCGTTTACTAGTTGCCAACG	Genome-specific PCR confirmation primer
red34_S	CAATGACCTTAGATTGGATTCAATGTTGTG	Genome-specific PCR confirmation primer
red35_As	GCAACATCGTCTCTTTTGCTTACG	Genome-specific PCR confirmation primer
red36_As_1	CGACCCTCGAAGCTAAGCTCCAGG	Genome-specific PCR confirmation primer
red36_As_2	CGTATCCAACGGTGACTCTTATCTGA	Genome-specific PCR confirmation primer
red36_S	GTGCGAAGAGCTTCCACATATTCATC	Genome-specific PCR confirmation primer
red37_As	TGGGAGGAAAACTTAAAACCCGAATCT	Genome-specific PCR confirmation primer
red37_S	CTAGACCCGATAATGTGACGACATTG	Genome-specific PCR confirmation primer
red38_As	AGAACGCGAAGATCTTGCAATTGTATC	Genome-specific PCR confirmation primer
red38_S	CAATATCTTCCTGGGGGGTTATACCAAC	Genome-specific PCR confirmation primer
red39_As	TCTCCACTGATTTTTTCAAAGCTATCCT	Genome-specific PCR confirmation primer
red39_S	AGGACGCTACGGCAAAAAAACGAG	Genome-specific PCR confirmation primer
red4_As	GCTTCTTCTCGCTATTGATCAATCAAGG	Genome-specific PCR confirmation primer
red40_As	CAGTAAGAGCTTTCATGGGAAGTCC	Genome-specific PCR confirmation primer
red40_S_1	CTACTACAATTATATCTACCTCTCAACACTG	Genome-specific PCR confirmation primer
red40_S_2	ATCTACCTCTCAACACTGGAATCCAA	Genome-specific PCR confirmation primer
red41_S	CTCCTGGTGTTGTCTTCTCGATATGC	Genome-specific PCR confirmation primer
red42_As	GAAGAAATGGCAAACCGGTGCTGAC	Genome-specific PCR confirmation primer

red42_As_i2	ACACTACCACATGCTCTTTTCTTCG	Genome-specific PCR confirmation primer
red42_S	GCATCATGGTTCTTACCCACATTCTTAGG	Genome-specific PCR confirmation primer
red42_S_i2	CGAACCGCAAGAAAGAGATCATGG	Genome-specific PCR confirmation primer
red44_As	CACGTAGGCACTAGCCTGATTCGC	Genome-specific PCR confirmation primer
red45_As	AACGAAGCTCTCACAAGTCTAATCTAC	Genome-specific PCR confirmation primer
red46_As	CAAGCCAATAAAATTCCCTAACATTAGCAAC	Genome-specific PCR confirmation primer
red46_As_i2	CAGAGAAGCAAAACCATCCAAGATGG	Genome-specific PCR confirmation primer
red46_As_i3	TGAGTTCATATGTGTAGATCAGCTGGTG	Genome-specific PCR confirmation primer
red46_As_i4	TGATTAGAAGTGTCAATGACTAACTCGCT	Genome-specific PCR confirmation primer
red46_S	ACGTGAGATGGTACGACATGTGGG	Genome-specific PCR confirmation primer
red46_S_i2	GATTGCTGGTTCTGCCTTTGACATG	Genome-specific PCR confirmation primer
red46_S_i3	GACTCAGGGAAAACTCAAGTTGGTTG	Genome-specific PCR confirmation primer
red47_As	CCGTTACATCGGTTTTGAATTCTTCGT	Genome-specific PCR confirmation primer
red47_As_i2	GCCAAGTACTTGCAGCTTATACATCC	Genome-specific PCR confirmation primer
red47_S	GTCTTTTCGTTGAACTTTTGATAGCACC	Genome-specific PCR confirmation primer
red48_S	TTAGCCCATTCGTAGACTTCCTCCG	Genome-specific PCR confirmation primer
red49_As	CAAAACTGCACCACGTAAATTGGTT	Genome-specific PCR confirmation primer
red49_As_i2	GCGAGCATTATGCTTGTATGGTTGATG	Genome-specific PCR confirmation primer
red49_S	TGGATCCATTGTTAGTCTAAGAAGCCG	Genome-specific PCR confirmation primer
red50_As	GTCGCCATGCACAAGTAACATCTTC	Genome-specific PCR confirmation primer
red51_As	CTGTGGCTAAACCTTTTGTTCTGCC	Genome-specific PCR confirmation primer
red51_As_i2	ACAGGCTTTTCAGAGAGACTTGGATC	Genome-specific PCR confirmation primer
red51_As_i3	TCTTAGCTGTCTCACCCACATGACC	Genome-specific PCR confirmation primer
red51_As_i4	CGGGACTCAGCTGAGAAGATAAGCA	Genome-specific PCR confirmation primer
red51_As_i5	CATCTCGATCATCTCCATCTTCAGTAG	Genome-specific PCR confirmation primer
red51_As_i6	GGTGGTGTGAGAATCAGATTCGATGTC	Genome-specific PCR confirmation primer
red51_As_i7	TGTTCCTCGATTTTACACTAGCTTGC	Genome-specific PCR confirmation primer
red51_As_i8	GCACTCTTGTCGCTAGATTCAACATC	Genome-specific PCR confirmation primer
red51_S	CCATTTACCGTGAATCCGACGCAC	Genome-specific PCR confirmation primer
red51_S_i2	ATAATCCACCACTAGCTAGAACTTATTGGT	Genome-specific PCR confirmation primer
red51_S_i3	CGTCGCTGCTTTGACGATAATCGTAAG	Genome-specific PCR confirmation primer
red51_S_i4	CATATTAATCGTCGCAAGCTAGTGCTC	Genome-specific PCR confirmation primer
red51_S_i5	CGATTTCCATTTACCGTGAATCCGA	Genome-specific PCR confirmation primer
red51_S_i6	CCATAAGCATAAGGTACATGAACCGCTG	Genome-specific PCR confirmation primer
red52_As	AAGGACATGTGAAATTGCTAATGCCAGTC	Genome-specific PCR confirmation primer
red52_As_i2	TTCTTGTGGATGGTTTCAAGAACTCAAC	Genome-specific PCR confirmation primer
red52_S	CCATCTTCCAGCCCCTCTAGAAGAAG	Genome-specific PCR confirmation primer
red53_As	TCAAAGAACGGAGAGGAACGAGTCG	Genome-specific PCR confirmation primer
red53_S	ATGCGGCTCATTACCACCATTCAC	Genome-specific PCR confirmation primer
red54_As	GAGATAATCGACGAGAAGCTTCGGGT	Genome-specific PCR confirmation primer
red54_S	CTTAGGTGCGATTTCATGAACTTTGC	Genome-specific PCR confirmation primer
red56_As	ACCTTGGAGACCTGATGCGGTTATG	Genome-specific PCR confirmation primer
red56_As_i2	CGCACTCAAGTCTTTACTCGAACGG	Genome-specific PCR confirmation primer
red56_S	CTGTGCTTGCTTTTTCCATGTGCTC	Genome-specific PCR confirmation primer
red56_S_i2	GTGGTGGTCCAAACTCCAAAGTCAC	Genome-specific PCR confirmation primer
red57_As	CTTGAATGAAGTTGAGATCGTGAGGG	Genome-specific PCR confirmation primer
red57_S	GTCAAAGAGTAACACCATATTGGACCG	Genome-specific PCR confirmation primer
red58_As	GTTACAGGCACATGCATGCCATTG	Genome-specific PCR confirmation primer
red59_As	GAATCTCAGACATGACTTGATCGCTAC	Genome-specific PCR confirmation primer
red59_S	GCGATCATGCATGACTCAAATTGG	Genome-specific PCR confirmation primer
red6_S	CCTGACAATAATATGCTTGGATGG	Genome-specific PCR confirmation primer
red60_S	GGCCGTTGTATCATTTATGTTTGTGAAAC	Genome-specific PCR confirmation primer
red61_As	TCACTGACTGGAACGATGTAGAAGGC	Genome-specific PCR confirmation primer
red61_S	CGATATACTTGACCATCCAATAATGGCTTG	Genome-specific PCR confirmation primer
red62_As	ACCAAGAAAAACACAGCTCTAGCCATC	Genome-specific PCR confirmation primer
red62_As_i2	TACCCCCATCTTCACCCGAGACTG	Genome-specific PCR confirmation primer
red62_As_i3	CCCCAATTAACCTTTAGAGACACAGAGG	Genome-specific PCR confirmation primer
red62_As_i4	GGAGTAGGTAGAGACGAGCTTTCACA	Genome-specific PCR confirmation primer
red62_As_i5	CATTGACATGACCGGGATGGAAACAC	Genome-specific PCR confirmation primer
red62_S	CGCTTTAGTGATTGTAACTGTCTAAGTGG	Genome-specific PCR confirmation primer
red63_S	AGGTCAGCTTCTAGGGACACCTGA	Genome-specific PCR confirmation primer
red64_As	AGGCTATTGTAGTACGGCTGGTGAC	Genome-specific PCR confirmation primer
red64_As_i2	AACCATGGAAAGTAGTTATGATGTGCC	Genome-specific PCR confirmation primer
red64_S	GTCGTCGATCTCTTCCAGATAATATTCA	Genome-specific PCR confirmation primer
red65_As	GTCCAGAGCTAACAGTGATCATTAACTCCT	Genome-specific PCR confirmation primer
red65_S	CGACACTCAACATTTTCCCGCCAA	Genome-specific PCR confirmation primer
red66_As	GTATGTCCTACTTGGAAACATATTTCAATTCC	Genome-specific PCR confirmation primer
red66_S	CGAAGTATGATTCTTTCAACGTGTGGGAC	Genome-specific PCR confirmation primer
red66_S_i2	GATGTACTCAGCTTCAGATACCTTCTGT	Genome-specific PCR confirmation primer
red70_As	GATCGCTTTGTAAAGTTTACTCCC	Genome-specific PCR confirmation primer
red71_As	TGAAGATTGTTAGGTCAGTGCGATA	Genome-specific PCR confirmation primer
red71_S	GAATCCATCAGCGACTTACAACAA	Genome-specific PCR confirmation primer

red72_S	GCTGAAGAGCTCTGTTGATCAACAAGTG	Genome-specific PCR confirmation primer
red74_S	GGTTCTTCTTCATCTTCTTCCTCTCCTTCC	Genome-specific PCR confirmation primer
red76_As	CTGATTTCTGTAAATAGTTGTGAGCTGAGACC	Genome-specific PCR confirmation primer
red76_As_i2	CAGAAACTGGCAAAACGGTGAAAACAACG	Genome-specific PCR confirmation primer
red77_As	GTTGCAGATCATACTGCTGATGATACTCC	Genome-specific PCR confirmation primer
red77_As_i2	CCTAGCCACGTCAGCCAATCTTGCTAC	Genome-specific PCR confirmation primer
red77_S	CTCGCGTCGCTTTTTGCTTCAGAATC	Genome-specific PCR confirmation primer
red77_S_i2	TTCGTCAGTTACAAAAGAAATTCGTCTGTATG	Genome-specific PCR confirmation primer
red78_As	TACGTGTAAAGCTGAGATCATTATTATCACAG	Genome-specific PCR confirmation primer
red79_As	GTTAGAACATACGGTATTGCAGACCGTCC	Genome-specific PCR confirmation primer
red79_S	CACTCTGCTTTCTCTTTGGTAATGCAATAGG	Genome-specific PCR confirmation primer
red79_S_i2	AGTGACCCGAAGAGAGTAAATTAGTTAGAG	Genome-specific PCR confirmation primer
red81_S	GAGTCGGTATCATATATGAGATTTTGGCATCC	Genome-specific PCR confirmation primer
red82_S	CCACATGATGATCCATCCATGATCGTAC	Genome-specific PCR confirmation primer
red83_As	GTAGAGACTAGTGGGTTTTGCACTAAGCTC	Genome-specific PCR confirmation primer
red83_S	GCCTCAATACCAAGTGAAGAGTCGATTC	Genome-specific PCR confirmation primer
Gm_1_S	TAATGTTGTCTAGGGGCTTCAGCGTC	Genome-specific PCR confirmation primer
Gm_1_As1	GAAGCCATCATCCATTGCACC	Genome-specific PCR confirmation primer
Gm_3_As	CATTCATTTGGGCTTTCCTATTCACAG	Genome-specific PCR confirmation primer
Gm_3_S	GGTGGGAAAGAGAAACACCTTTTCC	Genome-specific PCR confirmation primer
Os_3_As	GGCAAGATTTGACAGGCCTCATCA	Genome-specific PCR confirmation primer
Sl_2_As1	TGGTCTGATGATCTTGCTTCTTG	Genome-specific PCR confirmation primer
Sl_2_S1	CATGGGACTAATCTAGGTAAAGACA	Genome-specific PCR confirmation primer
Sl_4_As	GGATCGGCTCTTTACTCTACAGGTTAG	Genome-specific PCR confirmation primer
Sl_4_S	GAACTCTTCTCTCCAGTTGTTCAAGT	Genome-specific PCR confirmation primer
Sl_5_S	GACTCAAAACTAGCATTGCTAAAAGATGG	Genome-specific PCR confirmation primer
Sl_5_As1	AGCTAATGGCCTCGTGGTACC	Genome-specific PCR confirmation primer
Sl_7_As	AGCCTAATCCATTCAAATAACATGCCT	Genome-specific PCR confirmation primer
Sl_7_S1	GGTGGAGGTCCATTCTCATG	Genome-specific PCR confirmation primer
Sl_8_S	AAGAATAAAAGATGGCAATAATACCCCAC	Genome-specific PCR confirmation primer
St_8As	GCAAGAGCTTGATCTTCGGGACAC	Genome-specific PCR confirmation primer
Sl_8_As2	GACTTTCCCACTAACGATAGG	Genome-specific PCR confirmation primer
Zm_1As	TCGCCATACAAGAGCGAAGCCG	Genome-specific PCR confirmation primer
Zm_4S2	GTGCAACTGAAATCAAGCTTGGCTGC	Genome-specific PCR confirmation primer
Ta_1As1	CAGGAGTGGAACAAGCGTAG	Genome-specific PCR confirmation primer
Ta_1S3	TGAGCTTGCCGACACTATTC	Genome-specific PCR confirmation primer
Ta_4As1	AATAGTTGTCGCTACAGAGAGA	Genome-specific PCR confirmation primer
Ta_4As2	AACTAGGCGGAGCAGAGATAG	Genome-specific PCR confirmation primer
Ta_4S1	TCTCCACCATAAATACCTATGC	Genome-specific PCR confirmation primer
Ta_4S2	GATGCCGCAACACTTGACAA	Genome-specific PCR confirmation primer
Ta_4S4	ACGAGATGCGTTCCGTTCC	Genome-specific PCR confirmation primer
Ta_7As1	TGTGCTATGCTAATGACTGAGA	Genome-specific PCR confirmation primer
Ta_7S2	CGTCGCCCTATATATGGTACTC	Genome-specific PCR confirmation primer
Ta_10As1	TGCAGTGCTCCACTATGAAA	Genome-specific PCR confirmation primer
Ta_10S1	GTGGTGTGTACCTCGTTTGTC	Genome-specific PCR confirmation primer
Ta_13As1	ACTTGTGGGTTATCATTCGGTA	Genome-specific PCR confirmation primer
Ta_13As2	GCTCGAGCGAAGGCGGAGGTGGCG	Genome-specific PCR confirmation primer
Ta_13As3	ACTCGCGTGAGGTGGAATCCGTCG	Genome-specific PCR confirmation primer
Ta_13As4	CCCCGCCCGGCCATGCCCCGGTTC	Genome-specific PCR confirmation primer
Ta_13As5	TTCTCGTGTTCTTATCCCAC	Genome-specific PCR confirmation primer
Ta_13As6	ATTCGGTACTATGCTCGGTTAA	Genome-specific PCR confirmation primer
Ta_13S1	GTGTTCAGCACAGCGAACTA	Genome-specific PCR confirmation primer
Ta_13S2	CTTTCCAACGCCCACACACC	Genome-specific PCR confirmation primer
Ta_13S3	ATTCACGGCACCCCCGATAA	Genome-specific PCR confirmation primer
Ta_13S4	AGGTGAAATGTTGCACGTTC	Genome-specific PCR confirmation primer
Ta_13S5	CTAGTTCGCCCACACAACAC	Genome-specific PCR confirmation primer
Ta_16_S	AACGGCTGGGTGCTGTTCTTT	Genome-specific PCR confirmation primer
Ta_19_4S	TACAAAGATACGCAGGCCGAATA	Genome-specific PCR confirmation primer
Ta_19_As1	CTTGGCGGAATACGAGACC	Genome-specific PCR confirmation primer
Ta_22As3	TGCATTATGATCGCCTCTTGTT	Genome-specific PCR confirmation primer
Ta_22As5	TCAAGCGTGTCTTCTACGC	Genome-specific PCR confirmation primer
Ta_22S1	GATGTGTTTGATCTCCATGTCC	Genome-specific PCR confirmation primer
Ta_22S2	CATCACGCCTCACCTTCCTT	Genome-specific PCR confirmation primer
Ta_22S3	GTCGCCGAACACCATCACAG	Genome-specific PCR confirmation primer
Ta_22S4	GCACCAACAAGAGGGTGGTC	Genome-specific PCR confirmation primer
Ta_25S1	TACAGCATGCCAGCACTGAC	Genome-specific PCR confirmation primer
Ta_28As1	ACAGCAGCCCAGTGATACAT	Genome-specific PCR confirmation primer
Ta_28S1	ATCCCTGGCACCTAACAGAG	Genome-specific PCR confirmation primer
Ta_31S1	CAACTTGGATCGTGTGGATC	Genome-specific PCR confirmation primer

Supplementary Table 2. Summary of T-DNA integration sites in this study.
"Species" and "Sample": Species names and their independent transgenic lines; "Ref:Breakpoint", "RefSide", "tDNA", and "Direction": Characteristics of predicted TISs; "PCR P1" and "PCR P2": primer pairs used for TISs confirmation; "PCR-Sanger sequencing confirmed" and "Blast confirmed": TISs confirmed by PCR-sanger sequencing and BLAST analysis against reference genome; "Plasmid backbone": plasmid backbone used to generate the transgenic plants. "LBΔ" and "RBΔ" in multiple rows stand for truncated LB or RB regions.

Species	Sample	Ref:Breakpoint	RefSide	tDNA	Direction	PCR P1	PCR P2	PCR-Sanger sequencing confirmed	Blast confirmed	Plasmid backbone
Arabidopsis thaliana	red_2	1:22674352	Right	LB	Reverse	103LBpcr_As2	red2_As	Yes	Yes	pEarleyGate 103 (addgene #140007)
Arabidopsis thaliana	red_11	3:8817004	Left	LB	Forward	103LBpcr_As2	red11_S	Yes	Yes	pEarleyGate 103
Arabidopsis thaliana	red_12	1:22394492	Right	LB	Reverse	103LBpcr_As2	red12_3As	ND	-	pEarleyGate 103
Arabidopsis thaliana	red_12	2:3003031	Right	RB	Reverse	103RBpcr_As3	red12_3As_2	Yes	Yes	pEarleyGate 103
Arabidopsis thaliana	red_12	3:22147904	Left	LB	Forward	103LBpcr_As2	red12_S	Yes	Yes	pEarleyGate 103
Arabidopsis thaliana	red_12	3:22147929	Right	RB	Forward	103RBpcr_S2	Red12_As_i2	Yes	Yes	pEarleyGate 103
Arabidopsis thaliana	red_12	3:4310469	Left	LB	Forward	103LBpcr_As2	red12_S_i2	Yes	Yes	pEarleyGate 103
Arabidopsis thaliana	red_12	3:4310475	Right	LB	Reverse	103LBpcr_As2	Red12_As_i3	Yes	Yes	pEarleyGate 103
Arabidopsis thaliana	red_13	5:22156369	Left	RB	Reverse	103RBpcr_S2	red13_S	Yes	Yes	pEarleyGate 103
Arabidopsis thaliana	red_13	5:22156399	Right	LB	Reverse	103LBpcr_As2	red13_As	Yes	Yes	pEarleyGate 103
Arabidopsis thaliana	red_14	1:27669707	Left	LB	Forward	103LBpcr_As2	red14_S	Yes	Yes	pEarleyGate 103
Arabidopsis thaliana	red_14	1:27669706	Right	RB	Forward	103RBpcr_S2	Red14_As	Yes	Yes	pEarleyGate 103
Arabidopsis thaliana	red_15	1:22257685	Right	LB	Reverse	103LBpcr_As2	red15_As_1	Yes	Yes	pEarleyGate 103
Arabidopsis thaliana	red_15	1:22257686	Left	LB	Forward	103LBpcr_As2	red15_S_1	Yes	Yes	pEarleyGate 103
Arabidopsis thaliana	red_15	4:16442435	Left	RB	Reverse	103RBpcr_S2	red15_3S	ND	-	pEarleyGate 103
Arabidopsis thaliana	red_16	2:6695114	Left	LB	Reverse	103LBpcr_S3	red16_3S	ND	-	pEarleyGate 103
Arabidopsis thaliana	red_16	4:6922131	Left	RB	Reverse	103RBpcr_As2	151upAs	Yes	Yes	pEarleyGate 103
Arabidopsis thaliana	red_16	5:7520441	Left	LB	Forward	103LBpcr_As2	red16_S_i4	Yes	Yes	pEarleyGate 103
Arabidopsis thaliana	red_16	5:7520470	Right	LB	Reverse	103LBpcr_As2	red16_3As	Yes	Yes	pEarleyGate 103
Arabidopsis thaliana	red_16	5:10466012	Right	LB	Reverse	103LBpcr_As2	red16_As_i4	Yes	Yes	pEarleyGate 103
Arabidopsis thaliana	red_16	5:10465984	Left	LB	Forward	103LBpcr_As2	red16_3S_2	Yes	Yes	pEarleyGate 103
Arabidopsis thaliana	red_17	1:24367698	Right	LB	Reverse	103LBpcr_As2	red17_As	Yes	Yes	pEarleyGate 103
Arabidopsis thaliana	red_17	1:24368353	Right	LB	Reverse	103LBpcr_As2	red17_As_i2	Yes	Yes	pEarleyGate 103
Arabidopsis thaliana	red_19	1:8566430	Left	LB	Forward	103LBpcr_As2	red19_S	Yes	Yes	pEarleyGate 103
Arabidopsis thaliana	red_19	1:8566496	Right	RB	Forward	103RBpcr_S2	red19_3As	Yes	Yes	pEarleyGate 103
Arabidopsis thaliana	red_19	1:13626726	Right	LB	Reverse	103LBpcr_S5	red19_3As_3	ND	-	pEarleyGate 103
Arabidopsis thaliana	red_19	2:8603977	Right	LB	Reverse	103LBpcr_As2	red19_3As_5	ND	-	pEarleyGate 103
Arabidopsis thaliana	red_19	3:3173011	Right	RB	Forward	103RBpcr_S2	Red19_As	Yes	Yes	pEarleyGate 103
Arabidopsis thaliana	red_19	3:3172937	Left	LB	Forward	103LBpcr_As2	red19_S_i2	Yes	Yes	pEarleyGate 103
Arabidopsis thaliana	red_19	3:4560811	Right	RB	Reverse	103RBpcr_As4	red19_3As_4	ND	-	pEarleyGate 103
Arabidopsis thaliana	red_19	5:10710974	Right	LB	Forward	103LBpcr_S6	red19_3As_2	ND	-	pEarleyGate 103
Arabidopsis thaliana	red_20	3:16229926	Left	LB	Forward	103LBpcr_As2	red20_S	Yes	Yes	pEarleyGate 103
Arabidopsis thaliana	red_20	3:16229976	Right	LB	Reverse	103LBpcr_As2	red20_As	Yes	Yes	pEarleyGate 103
Arabidopsis thaliana	red_21	3:4848962	Left	LB	Forward	103LBpcr_As2	red21_S	Yes	Yes	pEarleyGate 103
Arabidopsis thaliana	red_22	1:10263392	Left	LB	Forward	103LBpcr_As2	red22_S	Yes	Yes	pEarleyGate 103
Arabidopsis thaliana	red_22	1:10263396	Right	LB	Reverse	103LBpcr_As2	red22_As_i2	Yes	Yes	pEarleyGate 103
Arabidopsis thaliana	red_22	3:6359255	Left	LB	Forward	103LBpcr_As2	red22_S_i2	Yes	Yes	pEarleyGate 103
Arabidopsis thaliana	red_22	3:6359279	Right	RB	Forward	103RBpcr_S2	red22_3As	Yes	Yes	pEarleyGate 103
Arabidopsis thaliana	red_23	5:6773881	Left	LB	Forward	103LBpcr_As2	red23_S	Yes	Yes	pEarleyGate 103
Arabidopsis thaliana	red_23	5:6773895	Right	LB	Reverse	103LBpcr_S2	33upAs	Yes	Yes	pEarleyGate 103
Arabidopsis thaliana	red_24	2:15173242	Right	LB	Reverse	103LBpcr_As2	red24_As	Yes	Yes	pEarleyGate 103
Arabidopsis thaliana	red_25	1:8533461	Left	LB	Forward	103LBpcr_As2	red25_S	Yes	Yes	pEarleyGate 103
Arabidopsis thaliana	red_25	1:8533517	Right	RB	Forward	103RBpcr_S2	red25_As	Yes	Yes	pEarleyGate 103
Arabidopsis thaliana	red_26	3:3415483	Left	LB	Forward	103LBpcr_As2	red26_S	Yes	Yes	pEarleyGate 103
Arabidopsis thaliana	red_26	3:3415495	Right	LB	Reverse	103LBpcr_As2	red26_As	Yes	Yes	pEarleyGate 103
Arabidopsis thaliana	red_27	1:4381725	Left	LB	Forward	103LBpcr_As2	red27_S	Yes	Yes	pEarleyGate 103
Arabidopsis thaliana	red_28	1:1123608	Right	LB	Reverse	103LBpcr_As2	red28_As	Yes	Yes	pEarleyGate 103
Arabidopsis thaliana	red_29	1:19094630	Right	LB	Reverse	103LBpcr_As2	red29_As	Yes	Yes	pEarleyGate 103
Arabidopsis thaliana	red_30	3:17207774	Right	LB	Reverse	103LBpcr_As2	red30_As	Yes	Yes	pEarleyGate 103
Arabidopsis thaliana	red_31	1:6181568	Right	LB	Reverse	103LBpcr_As2	red31_As	Yes	Yes	pEarleyGate 103
Arabidopsis thaliana	red_32	4:2740899	Right	LB	Reverse	103LBpcr_As2	red32_As	Yes	Yes	pEarleyGate 103
Arabidopsis thaliana	red_33	3:19186409	Right	RB	Forward	103RBpcr_S2	Red33_As	Yes	Yes	pEarleyGate 103
Arabidopsis thaliana	red_34	1:26030581	Right	LB	Reverse	103LBpcr_As2	red34_As	Yes	Yes	pEarleyGate 103
Arabidopsis thaliana	red_34	3:6604937	Left	LB	Forward	103LBpcr_As2	red34_S	Yes	Yes	pEarleyGate 103
Arabidopsis thaliana	red_35	2:8855325	Right	RB	Forward	103RBpcr_S2	red35_As	Yes	Yes	pEarleyGate 103
Arabidopsis thaliana	red_36	5:22375423	Left	LB	Forward	103LBpcr_As2	red36_S	Yes	Yes	pEarleyGate 103
Arabidopsis thaliana	red_36	5:22375468	Right	RB	Forward	103RBpcr_S2	Red36_As_1	Yes	Yes	pEarleyGate 103
Arabidopsis thaliana	red_37	4:8188814	Left	LB	Forward	103LBpcr_As2	red37_S	Yes	Yes	pEarleyGate 103
Arabidopsis thaliana	red_37	4:8188823	Right	RB	Forward	103RBpcr_S2	red37_As	Yes	Yes	pEarleyGate 103
Arabidopsis thaliana	red_38	5:1198362	Left	LB	Forward	103LBpcr_As2	red38_S	Yes	Yes	pEarleyGate 103
Arabidopsis thaliana	red_38	5:1198378	Right	LB	Reverse	103LBpcr_As2	red38_As	Yes	Yes	pEarleyGate 103
Arabidopsis thaliana	red_39	5:15733041	Left	LB	Forward	103LBpcr_As2	red39_S	Yes	Yes	pEarleyGate 103
Arabidopsis thaliana	red_39	5:15733072	Right	LB	Reverse	103LBpcr_As2	red39_As	Yes	Yes	pEarleyGate 103
Arabidopsis thaliana	red_40	5:16445417	Left	RB	Reverse	103RBpcr_S2	red40_S_1	Yes	Yes	pEarleyGate 103
Arabidopsis thaliana	red_40	5:16445419	Right	LB	Reverse	103LBpcr_As2	red40_As	Yes	Yes	pEarleyGate 103
Arabidopsis thaliana	red_41	2:7540866	Left	LB	Forward	103LBpcr_As2	red41_S	Yes	Yes	pEarleyGate 103
Arabidopsis thaliana	red_42	1:18247264	Left	LB	Forward	103LBpcr_As2	red42_S	Yes	Yes	pEarleyGate 103
Arabidopsis thaliana	red_42	1:18247319	Right	LB	Reverse	103LBpcr_As2	red42_As	Yes	Yes	pEarleyGate 103
Arabidopsis thaliana	red_42	5:21573701	Left	LB	Forward	103LBpcr_As2	red42_S_i2	Yes	Yes	pEarleyGate 103
Arabidopsis thaliana	red_42	5:21573714	Right	LB	Reverse	103LBpcr_As2	red42_As_i2	Yes	Yes	pEarleyGate 103
Arabidopsis thaliana	red_44	4:8147307	Right	LB	Reverse	103LBpcr_As2	red44_As	Yes	Yes	pEarleyGate 103
Arabidopsis thaliana	red_45	1:23342639	Right	LB	Forward	103LBpcr_As3	red45_3As	Yes	Yes	pEarleyGate 103
Arabidopsis thaliana	red_46	1:207668	Right	RB	Forward	103RBpcr_S3	red46_3As	ND	-	pEarleyGate 103

Arabidopsis thaliana	red_46	1:207662	Left	LB	Forward	103LBpcr_As3	red46_3S	ND	-	pEarleyGate 103
Arabidopsis thaliana	red_46	3:1301026	Left	LB	Forward	103LBpcr_As4	red46_3S_4	Yes	Yes	pEarleyGate 103
Arabidopsis thaliana	red_46	3:1301072	Right	LB	Reverse	103LBpcr_As3	red46_3As_2	ND	-	pEarleyGate 103
Arabidopsis thaliana	red_46	3:8551230	Right	LB	Reverse	103LBpcr_As2	red46_As_i4	Yes	Yes	pEarleyGate 103
Arabidopsis thaliana	red_46	3:8551199	Left	RB	Reverse	103RBpcr_S5	red46_3S_2	Yes	Yes	pEarleyGate 103
Arabidopsis thaliana	red_47	1:19913178	Left	LB	Forward	103LBpcr_As2	red47_S	Yes	Yes	pEarleyGate 103
Arabidopsis thaliana	red_47	1:19913187	Right	LB	Reverse	103LBpcr_As2	red47_As_i2	Yes	Yes	pEarleyGate 103
Arabidopsis thaliana	red_47	5:4591573	Right	LB	Reverse	103LBpcr_As2	red47_As	Yes	Yes	pEarleyGate 103
Arabidopsis thaliana	red_48	4:18316360	Left	LB	Forward	103LBpcr_As2	red48_S	Yes	Yes	pEarleyGate 103
Arabidopsis thaliana	red_49	5:2670137	Right	LB	Reverse	103LBpcr_As2	red49_As_i2	Yes	Yes	pEarleyGate 103
Arabidopsis thaliana	red_49	5:16412757	Right	LB	Reverse	103LBpcr_As2	red49_As	Yes	Yes	pEarleyGate 103
Arabidopsis thaliana	red_49	5:16412767	Left	LB	Forward	103LBpcr_As2	red49_S	Yes	Yes	pEarleyGate 103
Arabidopsis thaliana	red_50	4:18497246	Right	LB	Reverse	103LBpcr_As2	red50_As	Yes	Yes	pEarleyGate 103
Arabidopsis thaliana	red_51	1:7013314	Left	LB	Forward	103LBpcr_As2	red51_S	Yes	Yes	pEarleyGate 103
Arabidopsis thaliana	red_51	1:7013327	Right	LB	Reverse	103LBpcr_As2	red51_As_i5	Yes	Yes	pEarleyGate 103
Arabidopsis thaliana	red_51	2:12217670	Left	LB	Forward	103LBpcr_As2	red51_S_i2	Yes	Yes	pEarleyGate 103
Arabidopsis thaliana	red_51	2:12217704	Right	LB	Reverse	103LBpcr_As2	red51_As	Yes	Yes	pEarleyGate 103
Arabidopsis thaliana	red_51	2:12359150	Left	LB	Forward	103LBpcr_As2	red51_S_i3	Yes	Yes	pEarleyGate 103
Arabidopsis thaliana	red_51	2:12359181	Right	LB	Reverse	103LBpcr_As2	red51_As_i6	Yes	Yes	pEarleyGate 103
Arabidopsis thaliana	red_51	2:16808901	Right	LB	Reverse	103LBpcr_As2	red51_As_i7	Yes	Yes	pEarleyGate 103
Arabidopsis thaliana	red_51	2:16808910	Left	LB	Forward	103LBpcr_As2	red51_S_i4	Yes	Yes	pEarleyGate 103
Arabidopsis thaliana	red_51	3:1459237	Right	LB	Reverse	103LBpcr_As2	red51_As_i2	Yes	Yes	pEarleyGate 103
Arabidopsis thaliana	red_51	3:19206015	Right	RB	Forward	103RBpcr_S2	red51_As_i3	Yes	Yes	pEarleyGate 103
Arabidopsis thaliana	red_51	3:7052011	Right	LB	Reverse	103LBpcr_As2	red51_As_i4	Yes	Yes	pEarleyGate 103
Arabidopsis thaliana	red_52	1:2529231	Right	LB	Reverse	103LBpcr_As2	red52_As	Yes	Yes	pEarleyGate 103
Arabidopsis thaliana	red_52	5:13915947	Left	LB	Forward	103LBpcr_As2	red52_S	Yes	Yes	pEarleyGate 103
Arabidopsis thaliana	red_52	5:13915971	Right	LB	Reverse	103LBpcr_As2	red52_3As	Yes	Yes	pEarleyGate 103
Arabidopsis thaliana	red_53	1:15530834	Left	LB	Forward	103LBpcr_As2	red53_S	Yes	Yes	pEarleyGate 103
Arabidopsis thaliana	red_53	1:15530845	Right	LB	Reverse	103LBpcr_As2	red53_As	Yes	Yes	pEarleyGate 103
Arabidopsis thaliana	red_54	1:18097928	Right	LB	Reverse	103LBpcr_As5	red54_3As	ND	-	pEarleyGate 103
Arabidopsis thaliana	red_54	1:20530469	Right	LB	Reverse	103LBpcr_As5	red54_3As_2	ND	-	pEarleyGate 103
Arabidopsis thaliana	red_54	1:20537875	Left	RB	Reverse	103RBpcr_S2	red54_S	Yes	Yes	pEarleyGate 103
Arabidopsis thaliana	red_56	2:8080397	Left	LB	Forward	103LBpcr_As2	red56_S_i2	Yes	Yes	pEarleyGate 103
Arabidopsis thaliana	red_56	3:21028914	Left	LB	Forward	103LBpcr_As2	red56_S	Yes	Yes	pEarleyGate 103
Arabidopsis thaliana	red_56	3:21028831	Right	LB	Reverse	103LBpcr_As2	red56_As_i2	Yes	Yes	pEarleyGate 103
Arabidopsis thaliana	red_56	3:14201489	Right	LB	Reverse	103LBpcr_As2	red56_As	Yes	Yes	pEarleyGate 103
Arabidopsis thaliana	red_57	1:18097887	Left	LB	Forward	103LBpcr_As2	red57_S	Yes	Yes	pEarleyGate 103
Arabidopsis thaliana	red_57	2:12629910	Right	LB	Reverse	103LBpcr_As2	red57_As	Yes	Yes	pEarleyGate 103
Arabidopsis thaliana	red_58	3:11277255	Right	LB	Reverse	103LBpcr_As2	red58_As	Yes	Yes	pEarleyGate 103
Arabidopsis thaliana	red_59	3:66304	Right	LB	Reverse	103LBpcr_As2	red59_As	Yes	Yes	pEarleyGate 103
Arabidopsis thaliana	red_60	1:20852451	Left	LB	Forward	103LBpcr_As2	red60_S	Yes	Yes	pEarleyGate 103
Arabidopsis thaliana	red_61	2:16748008	Left	LB	Forward	103LBpcr_As2	red61_S	Yes	Yes	pEarleyGate 103
Arabidopsis thaliana	red_61	2:17635022	Right	LB	Reverse	103LBpcr_As2	red61_As	Yes	Yes	pEarleyGate 103
Arabidopsis thaliana	red_62	2:717068	Right	LB	Reverse	103LBpcr_As2	red62_As_i2	Yes	Yes	pEarleyGate 103
Arabidopsis thaliana	red_62	3:10152028	Right	LB	Reverse	103LBpcr_As2	red62_As_i3	Yes	Yes	pEarleyGate 103
Arabidopsis thaliana	red_62	5:12628423	Right	LB	Reverse	103LBpcr_As2	red62_As_i4	Yes	Yes	pEarleyGate 103
Arabidopsis thaliana	red_62	5:6612928	Left	LB	Forward	103LBpcr_As2	red62_S	Yes	Yes	pEarleyGate 103
Arabidopsis thaliana	red_62	5:6612973	Right	LB	Reverse	103LBpcr_As2	red62_As_i5	Yes	Yes	pEarleyGate 103
Arabidopsis thaliana	red_63	5:3252818	Left	LB	Forward	103LBpcr_As2	red63_S	Yes	Yes	pEarleyGate 103
Arabidopsis thaliana	red_64	2:113189	Right	RB	Forward	103RBpcr_S3	red64_3As_2	Yes	Yes	pEarleyGate 103
Arabidopsis thaliana	red_64	3:20476801	Left	LB	Forward	103LBpcr_As2	red64_S	Yes	Yes	pEarleyGate 103
Arabidopsis thaliana	red_64	3:20476824	Right	LB	Reverse	103LBpcr_As2	red64_3As	Yes	Yes	pEarleyGate 103
Arabidopsis thaliana	red_65	1:17268255	Left	LB	Forward	103LBpcr_As2	red65_S	Yes	Yes	pEarleyGate 103
Arabidopsis thaliana	red_65	1:17268267	Right	LB	Reverse	103LBpcr_As2	red65_As	Yes	Yes	pEarleyGate 103
Arabidopsis thaliana	red_66	2:8825493	Right	RB	Forward	103RBpcr_S2	Red66_As	Yes	Yes	pEarleyGate 103
Arabidopsis thaliana	red_66	2:8825474	Left	LB	Forward	103LBpcr_As2	red66_3S	Yes	Yes	pEarleyGate 103
Arabidopsis thaliana	red_66	5:3797755	Left	RB	Reverse	103RBpcr_S2	red66_3S_2	Yes	Yes	pEarleyGate 103
Arabidopsis thaliana	red_70	4:14096027	Right	LB	Reverse	103LBpcr_As2	Red70_As	Yes	Yes	pEarleyGate 103
Arabidopsis thaliana	red_71	4:8173310	Left	LB	Forward	103LBpcr_As2	Red71_S	Yes	Yes	pEarleyGate 103
Arabidopsis thaliana	red_71	4:8173323	Right	RB	Forward	103RBpcr_S2	red71_As	Yes	Yes	pEarleyGate 103
Arabidopsis thaliana	red_72	5:4851702	Left	LB	Forward	103LBpcr_As2	red72_S	Yes	Yes	pEarleyGate 103
Arabidopsis thaliana	red_74	5:1390803	Left	LB	Forward	103LBpcr_As2	red74_S	Yes	Yes	pEarleyGate 103
Arabidopsis thaliana	red_76	3:6174244	Right	RB	Forward	103RBpcr_S2	red76_As	Yes	Yes	pEarleyGate 103
Arabidopsis thaliana	red_76	5:20176019	Right	LB	Reverse	103LBpcr_As2	red76_3As	Yes	Yes	pEarleyGate 103
Arabidopsis thaliana	red_77	1:10029845	Left	LB	Forward	103LBpcr_As2	red77_S	Yes	Yes	pEarleyGate 103
Arabidopsis thaliana	red_77	1:10029873	Right	LB	Reverse	103LBpcr_As2	red77_As	Yes	Yes	pEarleyGate 103
Arabidopsis thaliana	red_77	3:15987469	Right	RB	Forward	103RBpcr_S2	red77_As_i2	Yes	Yes	pEarleyGate 103
Arabidopsis thaliana	red_77	3:8110063	Left	LB	Forward	103LBpcr_As2	red77_S_i2	Yes	Yes	pEarleyGate 103
Arabidopsis thaliana	red_78	1:692794	Right	LB	Reverse	103LBpcr_As2	red78_As	Yes	Yes	pEarleyGate 103
Arabidopsis thaliana	red_79	1:23902403	Left	LB	Forward	103LBpcr_As2	red79_S	Yes	Yes	pEarleyGate 103
Arabidopsis thaliana	red_79	1:8566496	Right	RB	Forward	103RBpcr_S3	red79_3As	ND	-	pEarleyGate 103
Arabidopsis thaliana	red_79	3:6359279	Right	RB	Forward	103RBpcr_S6	red79_3As_2	ND	-	pEarleyGate 103
Arabidopsis thaliana	red_81	1:10752298	Left	LB	Forward	103LBpcr_S2	126upAs	Yes	Yes	pEarleyGate 103
Arabidopsis thaliana	red_82	4:17159634	Left	LB	Forward	103LBpcr_As2	red82_S	Yes	Yes	pEarleyGate 103
Arabidopsis thaliana	red_83	1:11219348	Left	LB	Forward	103LBpcr_As2	red83_S	Yes	Yes	pEarleyGate 103
Arabidopsis thaliana	red_83	1:11219375	Right	LB	Reverse	103LBpcr_As2	red83_As	Yes	Yes	pEarleyGate 103
Oryza sativa	OS_2	Chr12:22341429	Left	LB	Forward	1300LBpcr_As2	OS_2_S	Yes	Yes	pCambia1300
Oryza sativa	OS_2	Chr12:22341461	Right	RB	Forward	1300RBpcr_S2	OS_2_As	Yes	Yes	pCambia1300
Oryza sativa	OS_3	Chr11:5788552	Left	LB	Forward	1300LBpcr_As2	OS_3_S	Yes	Yes	pCambia1300
Oryza sativa	OS_3	Chr11:5788592	Right	RB	Forward	1300RBpcr_S2	OS_3_As	Yes	Yes	pCambia1300
Oryza sativa	OS_4	Chr6:26503230	Left	LB	Forward	1300LBpcr_As2	OS_4_S	Yes	Yes	pCambia1300
Oryza sativa	OS_4	Chr6:26503287	Right	LB	Reverse	1300LBpcr_As2	OS_4_As	Yes	Yes	pCambia1300

Oryza sativa	OS_5	Chr1:23983642	Left	LB	Forward	1300LBpcr_As2	OS_5_S	Yes	Yes	pCambia1300
Oryza sativa	OS_5	Chr1:23983660	Right	LB	Reverse	1300LBpcr_As2	OS_5_As	Yes	Yes	pCambia1300
Oryza sativa	OS_6	Chr1:29923711	Left	LB	Forward	1300LBpcr_As2	OS_6_S	Yes	Yes	pCambia1300
Oryza sativa	OS_6	Chr1:29923781	Right	LB	Reverse	1300LBpcr_As2	OS_6_As	Yes	Yes	pCambia1300
Oryza sativa	OS_7	Chr4:23065689	Left	LB	Forward	1300LBpcr_As2	OS_7_S	Yes	Yes	pCambia1300
Oryza sativa	OS_7	Chr4:23065706	Right	RB	Forward	1300RBpcr_S2	OS_7_As	Yes	Yes	pCambia1300
Oryza sativa	OS_8	Chr1:29923711	Left	LB	Forward	1300LBpcr_As2	OS_8_S	Yes	Yes	pCambia1300
Oryza sativa	OS_8	Chr1:29923781	Right	LB	Reverse	1300LBpcr_As2	OS_8_As	Yes	Yes	pCambia1300
Oryza sativa	OS_9	Chr12:5206143	Right	LB	Reverse	1300LBpcr_As2	OS_9_As	Yes	Yes	pCambia1300
Oryza sativa	OS_10	Chr4:19194796	Left	LB	Forward	1300LBpcr_As2	OS_10_S	Yes	Yes	pCambia1300
Oryza sativa	OS_10	Chr4:19194819	Right	LB	Reverse	1300LBpcr_As2	OS_10_As	Yes	Yes	pCambia1300
Oryza sativa	OS_11	Chr2:4517882	Left	LB	Forward	1300LBpcr_As2	OS_12_S1	Yes	Yes	pCambia1300
Oryza sativa	OS_11	Chr2:4517966	Right	RB	Forward	1300RBpcr_S2	OS_12_As	Yes	Yes	pCambia1300
Oryza sativa	OS_11	Chr3:13201239	Left	RB	Reverse	1300RBpcr_S2	OS_12_S2	Yes	Yes	pCambia1300
Solanum lycopersicum	Sl_2	2:43845454	Left	LB	Forward	103LBpcr_As2	SI_2f1	Yes	Yes	pEarleyGate 103
Solanum lycopersicum	Sl_2	2:43847281	Right	LB	Reverse	103LBpcr_As2	SI_2r1	Yes	Yes	pEarleyGate 103
Solanum lycopersicum	Sl_4	1:91229410	Right	LB	Reverse	103LBpcr_As2	Sl_4_As	Yes	Yes	pEarleyGate 103
Solanum lycopersicum	Sl_4	1:91229347	Left	RB	Reverse	103RBpcr_S2	Sl_4_S	Yes	Yes	pEarleyGate 103
Solanum lycopersicum	Sl_5	6:45373853	Left	RB	Reverse	103RBpcr_S2	Sl_5_S	Yes	Yes	pEarleyGate 103
Solanum lycopersicum	Sl_5	6:45373916	Right	LBΔ	Reverse	Ocster_As	Sl_5_As1	Yes	Yes	pEarleyGate 103
Solanum lycopersicum	Sl_7	6:39432635	Right	RB	Forward	103RBpcr_S2	Sl_7_As	Yes	Yes	pEarleyGate 103
Solanum lycopersicum	Sl_7	6:39432475	Left	LBΔ	Forward	Hyg_As	Sl_7_S1	Yes	Yes	pEarleyGate 103
Solanum lycopersicum	Sl_8	5:22716039	Left	RB	Reverse	103RBpcr_S2	Sl_8_S	Yes	Yes	pEarleyGate 103
Solanum lycopersicum	Sl_8	5:22716304	Right	LBΔ	Reverse	Ocster_As	Sl_8_As2	Yes	Yes	pEarleyGate 103
Glycine max	Gm_1	Gm03:33929443	Left	LB	Forward	103LBpcr_As2	Gm_1_S	Yes	Yes	pEarleyGate 103
Glycine max	Gm_1	Gm03:33929460	Right	RB	Forward	Ocster_S	Gm_1_As1	Yes	Yes	pEarleyGate 103
Glycine max	Gm_3	Gm02:2588786	Left	LB	Forward	103LBpcr_As2	Gm_3_S	Yes	Yes	pEarleyGate 103
Glycine max	Gm_3	Gm02:2588795	Right	LB	Reverse	103LBpcr_As2	Gm_3_As	Yes	Yes	pEarleyGate 103
Zea mays	Zm_1	2:44992367	Right	LB	Reverse	1300LBpcr_As2	Zm_1As	Yes	Yes	pCambia1300
Zea mays	Zm_4	3:233961984	Left	LB	Forward	1300LBpcr_As2	Zm_4S2	Yes	Yes	pCambia1300
Solanum tuberosum	St_8	chr01:1429743	Right	RB	Forward	PPL_RBpcr_S2	St_8As	Yes	Yes	PPL
Triticum aestivum	Ta_1	chr2A:793385679	Right	RB	Forward	pBUERBpcr_S3	Ta_1S2	Yes	Yes	pBUE411(pCambia)
Triticum aestivum	Ta_1	chr2D:631820938	Left	LB	Forward	pBUELBpcr_As3	Ta_1S3	Yes	Yes	pBUE411(pCambia)
Triticum aestivum	Ta_4	chr4A:37283953	Right	RB	Forward	pBUERBpcr_S3	Ta_4As1	Yes	Yes	pBUE411(pCambia)
Triticum aestivum	Ta_4	chr4A:37283827	Left	LB	Forward	pBUELBpcr_As3	Ta_4S4	Yes	Yes	pBUE411(pCambia)
Triticum aestivum	Ta_4	chr6B:639482700	Right	RB	Forward	pBUERBpcr_S3	Ta_4As2	Yes	Yes	pBUE411(pCambia)
Triticum aestivum	Ta_4	chr6B:639482678	Left	RB	Reverse	pBUERBpcr_S3	Ta_4S1	Yes	Yes	pBUE411(pCambia)
Triticum aestivum	Ta_4	chr6B:72999626	Left	RB	Reverse	pBUERBpcr_S3	Ta_4S2	Yes	Yes	pBUE411(pCambia)
Triticum aestivum	Ta_4	chr6B:72999687	Right	RB	Reverse	pBUERBpcr_As3	Ta_4As4	Yes	Yes	pBUE411(pCambia)
Triticum aestivum	Ta_7	chr4A:498425330	Left	LB	Forward	pBUELBpcr_As3	Ta_7S2	Yes	Yes	pBUE411(pCambia)
Triticum aestivum	Ta_7	chr4A:498425465	Right	RB	Forward	pBUERBpcr_S3	Ta_7As1	Yes	Yes	pBUE411(pCambia)
Triticum aestivum	Ta_10	chr6B:494346052	Left	RB	Reverse	pBUERBpcr_S3	Ta_10S1	Yes	Yes	pBUE411(pCambia)
Triticum aestivum	Ta_10	chr6B:494346443	Right	RB	Forward	pBUERBpcr_S3	Ta_10As1	Yes	Yes	pBUE411(pCambia)
Triticum aestivum	Ta_13	chr6A:561496741	Right	LB	Reverse	pBUELBpcr_As3	Ta_13As6	Yes	Yes	pBUE411(pCambia)
Triticum aestivum	Ta_13	chr6A:561496741	Left	LB	Forward	pBUELBpcr_As3	Ta_13S2	Yes	Yes	pBUE411(pCambia)
Triticum aestivum	Ta_16	chr7D:53408824	Left	LB	Forward	pBUELBpcr_As2	Ta18_S	Yes	Yes	pBUE411(pCambia)
Triticum aestivum	Ta_19	chr1B:30173060	Left	LB	Forward	BlpR_2As	Ta19_4S	Yes	Yes	pBUE411(pCambia)
Triticum aestivum	Ta_19	chr1B:30173104	Right	LB	Reverse	BlpR_2As	Ta_19_As1	Yes	Yes	pBUE411(pCambia)
Triticum aestivum	Ta_22	chr2B:527710671	Left	RB	Reverse	pBUERBpcr_S3	Ta_22S2	Yes	Yes	pBUE411(pCambia)
Triticum aestivum	Ta_22	chr2B:527711596	Right	RB	Forward	pBUERBpcr_S3	Ta_22As3	Yes	Yes	pBUE411(pCambia)
Triticum aestivum	Ta_22	chr4A:656361197	Left	RB	Reverse	pBUERBpcr_S3	Ta_22S3	Yes	Yes	pBUE411(pCambia)
Triticum aestivum	Ta_22	chr4A:656361222	Right	RBΔ	Reverse	pBUERBpcr_As3	Ta_22As5	Yes	Yes	pBUE411(pCambia)
Triticum aestivum	Ta_22	chr2A:647236162	Left	RB	Reverse	pBUERBpcr_S3	Ta_22S4	Yes	Yes	pBUE411(pCambia)
Triticum aestivum	Ta_25	chr4D:513077658	Left	LB	Forward	pBUELBpcr_As3	Ta_25S1	Yes	Yes	pBUE411(pCambia)
Triticum aestivum	Ta_28	chr4B:408235032	Left	LB	Forward	pBUELBpcr_As3	Ta_28S1	Yes	Yes	pBUE411(pCambia)
Triticum aestivum	Ta_28	chr4B:408235043	Right	RB	Forward	pBUERBpcr_S3	Ta_28As1	Yes	Yes	pBUE411(pCambia)
Triticum aestivum	Ta_31	chr7B:23740277	Left	LB	Forward	pBUELBpcr_As3	Ta_31S1	Yes	Yes	pBUE411(pCambia)

Supplementary Table 3. Predicted GSH region in Arabidopsis.

Chromasome	Start	End	Length (bp)
Chr1	11025913	11026179	266
Chr1	12673870	12675199	1329
Chr1	12720423	12726091	5668
Chr1	12835680	12841996	6316
Chr1	13239796	13243397	3601
Chr1	13716498	13739353	22855
Chr1	14613904	14633259	19355
Chr1	15630468	15640171	9703
Chr1	15778026	15795110	17084
Chr1	15993328	16017111	23783
Chr1	16167911	16168982	1071
Chr1	16288796	16298881	10085
Chr1	16329234	16335973	6739
Chr1	16952524	16953143	619
Chr1	17210735	17213554	2819
Chr2	1810392	1813239	2847
Chr2	2634902	2637990	3088
Chr2	2670715	2688170	17455
Chr2	2790691	2796624	5933
Chr2	2826349	2832352	6003
Chr2	4030773	4071832	41059
Chr2	4817319	4821866	4547
Chr2	5896193	5896549	356
Chr2	6068524	6078338	9814
Chr2	6121918	6133072	11154
Chr2	6333397	6345181	11784
Chr2	10643663	10643708	45
Chr3	11660839	11661740	901
Chr3	11850502	11867865	17363
Chr3	11898712	11899118	406
Chr3	12156502	12157766	1264
Chr3	12282874	12285923	3049
Chr3	12469304	12478977	9673
Chr3	12610432	12614436	4004
Chr3	12745571	12758983	13412
Chr3	12783539	12785860	2321
Chr3	12858857	12865070	6213
Chr3	12887422	12897876	10454
Chr3	12920391	12927891	7500
Chr3	13019893	13022457	2564
Chr3	13250154	13266081	15927
Chr3	13287155	13292212	5057
Chr3	13333052	13346994	13942
Chr3	14471497	14474034	2537
Chr3	14917035	14919713	2678

Chr3	15041152	15065839	24687
Chr3	15380059	15381567	1508
Chr3	15549418	15555223	5805
Chr4	1626192	1639664	13472
Chr4	1984014	2010435	26421
Chr4	2146249	2149000	2751
Chr4	3649710	3653484	3774
Chr4	3777611	3785380	7769
Chr4	4325510	4335347	9837
Chr4	4655338	4681761	26423
Chr4	4702653	4710248	7595
Chr5	10112274	10125433	13159
Chr5	10149013	10152782	3769
Chr5	10407486	10419800	12314
Chr5	10442357	10444392	2035
Chr5	10465807	10467770	1963
Chr5	10510179	10515077	4898
Chr5	11008783	11012082	3299
Chr5	11498280	11531462	33182
Chr5	12575757	12578837	3080
Chr5	13073379	13078326	4947
Chr5	13501673	13507886	6213
Chr5	13535791	13539342	3551
Chr5	13560124	13574105	13981
Chr5	14506849	14508315	1466

Supplementary Table 4 . A cost estimation for Tn5-mediated T-LOC, TAIL-PCR and WGS-based T-LOC.

	**Tn5-mediated T-LOC ($/sample)**	**TAIL-PCR ($/sample)**	**WGS-based T-LOC ($/sample)**
Primers	0.10	0.03	0.03
Tn5 transposase	5.6 (commerical);or ¥ 0.56 (home-made)	NA	NA
High-ferdelity DNA polymerase with strand replacement activity for NGS library construction	0.9	NA	NA
NGS library construction	NA	NA	15
High-ferdelity DNA polymerase for PCR confirmation	0.9	1.35	0.9
SPRI-based DNA clean beads	0.69	0.69	0.69
Library QC	0.41	NA	4.86
NGS sequencing ($2.5/G raw data)	2.5	NA	29 (rice);75 (soybean);1162 (wheat) at 30× coverages
Sanger sequencing ($1.4/reacion)	2.8	2.8	2.8
**Total cost ($)/sample**	9 or 14	5	51 for rice; 97 for soybean; 1184 for wheat

Supplementary Table 5. Sequences of LB and RB regions in T-DNA backbone in this study.

The underlined sequences are T-DNA left border (LB) or right border (RB) repeats; the sequncs highlighted in blue are part of bridge primers for TTLOC.

Backbone vector	LB region (500 bp)	RB region (500 bp)
pEarleyGate 103	TGGCAGGATATATTGTGGTGTAAACAAATTGACGCTTAGACAACTTAATA<br>ACACATTGCGGACGTTTTTAATGTACTGAATTAACGCCGAATTAATTCGA<br>GCTCGGATCTGATAATTTATTTGAAAATTCATAAGAAAAGCAAACGTTAC<br>ATGAATTGATGAAACAATACAAAGACAGATAAAGCCACGCACATTTAGG<br>ATATTGGCCGAGATTACTGAATATTGAGTAAGATCACGGAATTTCTGACA<br>GGAGCATGTCTTCAATTCAGCCCAAATGGCAGTTGAAATACTCAAACCGC<br>CCCATATGCAGGAGCGGATCATTCATTGTTTGTTTGGTTGCCTTTGCCAAC<br>ATGGGAGTCCAAGATTCTGCAGTCAAATCTCGGTGACGGGCAGGACCGGA<br>CGGGGCGGTACCGGCAGGCTGAAGTCCAGCTGCCAGAAACCCACGTCATG<br>CCAGTTCCCGTGCTTGAAGCCGGCCGCCCGCAGCATGCCGCGGGGGGCAT	GTGAGATTCCTTGAAGTTGAGTATTGGCCGTCCGCTCTACCGAAAGTTAC<br>GGGCACCATTCAACCCGGTCCAGCACGGCGGCCGGGTAACCGACTTGCTG<br>CCCCGAGAATTATGCAGCATTTTTTTGGTGTATGTGGGCCCCAAATGAAG<br>TGCAGGTCAAACCTTGACAGTGACGACAAATCGTTGGGCGGGTCCAGGGC<br>GAATTTTGCGACAACATGTCGAGGCTCAGCAGGACCTGCAGGCATGCAAG<br>CTTGGCACTGGCCGTCGTTTTACAACGTCGTGACTGGGAAAACCCTGGCG<br>TTACCCAACTTAATCGCCTTGCAGCACATCCCCCTTTCGCCAGCTGGCGTA<br>ATAGCGAAGAGGCCCGCACCGATCGCCCTTCCCAACAGTTGCGCAGCCTG<br>AATGGCGAATGCTAGAGCAGCTTGAGCTTGGATCAGATTGTCGTTTCCCG<br>CCTTCAGTTTAAACTATCAGTGTTTGACAGGATATATTGGCGGGTAAAC
pEarleyGate 103_hyg	TGGCAGGATATATTGTGGTGTAAACAAATTGACGCTTAGACAACTTAATA<br>ACACATTGCGGACGTTTTTAATGTACTGAATTAACGCCGAATTCGATAAT<br>TTATTTGAAAATTCATAAGAAAAGCAAACGTTACATGAATTGATGAAAC<br>AATACAAAGACAGATAAAGCCACGCACATTTAGGATATTGGCCGAGATT<br>ACTGAATATTGAGTAAGATCACGGAATTTCTGACAGGAGCATGTCTTCAA<br>TTCAGCCCAAATGGCAGTTGAAATACTCAAACCGCCCCATATGCAGGAGC<br>GGATCATTCATTGTTTGTTTGGTTGCCTTTGCCAACATGGGAGTCCAAGAT<br>CCTATTCCTTTGCCCTCGGACGAGTGCTGGGGCGTCGGTTTCCACTATCGG<br>CGAGTACTTCTACACAGCCATCGGTCCAGACGGCCGCGCTTCTGCGGGCG<br>ATTTGTGTACGCCCGACAGTCCCGGCTCCGGATCGGACGATTGCGTCGCAT<br>CGA	GTGAGATTCCTTGAAGTTGAGTATTGGCCGTCCGCTCTACCGAAAGTTAC<br>GGGCACCATTCAACCCGGTCCAGCACGGCGGCCGGGTAACCGACTTGCTG<br>CCCCGAGAATTATGCAGCATTTTTTTGGTGTATGTGGGCCCCAAATGAAG<br>TGCAGGTCAAACCTTGACAGTGACGACAAATCGTTGGGCGGGTCCAGGGC<br>GAATTTTGCGACAACATGTCGAGGCTCAGCAGGACCTGCAGGCATGCSSac<br>GGCACTGGCCGTCGTTTTACAACGTCGTGACTGGGAAAACCCTGGCGTTA<br>CCCAACTTAATCGCCTTGCAGCACATCCCCCTTTCGCCAGCTGGCGTAATA<br>GCGAAGAGGCCCGCACCGATCGCCCTTCCCAACAGTTGCGCAGCCTGAAT<br>GGCGAATGCTAGAGCAGCTTGAGCTTGGATCAGATTGTCGTTTCCCGCCT<br>TCAGTTTAAACTATCAGTGTTTGACAGGATATATTGGCGGGTAAAC
pCambia1300	tggcaggatatattgtggtgtaaacaaattgacgcttagacaacttaataacacattgcggacgtttttaatgttagactga<br>attaacgccgaattaattcggggggatctggattttagtactggatttggttttaggaattagaaattttattgatagaagtattt<br>tacaaatacaaatacatactaagggtttcttatatgctcaacacatgagcgaaaccctataggaaccctaattcccttatctg<br>ggaactactcacacattattatggagaaactcgagcttgtcgatcgacagatccggtcggcatctactctatttctttgccc<br>tcggacgagtgctggggcgtcggtttccactatcggcgagtacttctacacagccatcggtccagacggccgcgcttc<br>tgcgggcgatttgtgtacgcccgacagtcccggctccggatcggacgattgcgtcgcatcgaccctgcgcccaagct<br>gcatcatcgaaa	tgtcatctatgttactagatcgggccatccgcactgtagcggatggcctaaaaaaaaaactagaagagacgagtctga<br>gactcagcgtctcggtcgcagtcataacttcgtatagcatacattatacgaagttatgggccgcattaccctgttatcccta<br>ggccgcataacttcgtatagcctacattataggatggagggatatcctctcttaaggtagcgagcaagctctaagagga<br>gtgtcgacaagcttggcactggccgtcgttttacaacgtcgtgactgggaaaaccctggcgttacccaacttaatcgcct<br>tgcagcacatccccctttcgccagctggcgtaatagcgaagaggcccgcaccgatcgcccttcccaacagttgcgca<br>gcctgaatggcgaatgctagagcagcttgagcttggatcagattgtcgtttcccgccttcagtttaaactatcagtgtttga<br>caggatatattggcgggtaaac
PPL	tggcaggatatattgtggtgtaaacaaattgacgcttagacaacttaataacacattgcggacgtttttaatgtactgaatta<br>acgccgaattgaattatcagcttgcatgccggtcGATCTAGTAACATAGATGACACCGCGCGC<br>GATAATTTATCCTAGTTTGCGCGCTATATTTTGTTTTCTATCGCGTATTAA<br>ATGTATAATTGCGGGACTCTAATCATAAAAACCCATCTCATAAATAACGT<br>CATGCATTACATGTTAATTATTACATGCTTAACGTAATTCAACAGAAATT<br>ATATGATAATCATCGCAAGACCGGCAACAGGATTCAATCTTAAGAAACTT<br>TATTGCCAAATGTTTGAACGATCTGCTTGACTCTAGCTAGAGTCCGAACCC<br>CAGAGTCCCGCTCAGAAGAACTCGTCAAGAAGGCGATAGAAGGCGATGC<br>GCTGCGAATCGGGAGCGGCGATACCGTAAAGCACGAGGAAGCGGTCAGC<br>CCAT	CTGGGCAATGGAATCCGAGGAGGTTTCCCGATATTACCCTTTGTTGAAAA<br>GTCTCAATAGCCCTTTGGTCTTCTGAGACTGTATCTTTGATATTCTTGGAG<br>TAGACGAGAGTGTCGTGCTCCACCATGTTCACATCAATCCACTTGCTTTGA<br>AGACGTGGTTGGAACGTCTTCTTTTTCCACGATGCTCCTCGTGGGTGGGG<br>GTCCATCTTTGGGACCACTGTCGGCAGAGGCATCTTGAACGATAGCCTTTC<br>CTTTATCGCAATGATGGCATTTGTAGGTGCCACCTTCCTTTTCTACTGTCC<br>TTTTGATGAAGTGACAGATAGCTGGGCAATGGAATCCGAGGAGGTTTCCC<br>GATATTACCCTTTGTTGAAAAGTCTCACCCAAGCTTGGGCAAATGGTGTT<br>GTTAATTAAGAGCTTAGCTTGAGCTTGGATCAGATTGTCGTTTCCCGCCTT<br>CAGTTTAAACTATCAGTGTTTGACAGGATATATTGGCGGGTAAAC
pBUE411(pCambia)	TGGCAGGATATATTGTGGTGTAAACAAATTGACGCTTAGACAACTTAATA<br>ACACATTGCGGACGTTTTTAATGTACTGAATTAACGCCGAATTAATTCGG<br>GGGATCTGGATTTTAGTACTGGATTTTGGTTTTAGGAATTAGAAATTTTA<br>TTGATAGAAGTATTTTACAAATACAAATACATACTAAGGGTTTCTTATAT<br>GCTCAACACATGAGCGAAACCCTATAGGAACCCTAATTCCCTTATCTGGG<br>AACTACTCACACATTATTATGGAGAAACTCGAGTCAAATCTCGGTGACGG<br>GCAGGACCGGACGGGGCGGTACCGGCAGGCTGAAGTCCAGCTGCCAGAA<br>ACCCACGTCATGCCAGTTCCCGTGCTTGAAGCCGGCCGCCCGCAGCATGCC<br>GCGGGGGGCATATCCGAGCGCCTCGTGCATGCGCACGCTCGGGTCGTTGG<br>GCAGCCCGATGACAGCGACCACGCTCTTGAAGCCCTGTGCCTCCAGGGAC	tttccatttttgttactaataacagttcagaagctcttctcaaatgtcagtatctacttgtcaattattatttctcacaaattatcagc<br>acgctagtcaatgttaaaactctccctgccgccaactataacagcaattgtgcaggccaggcaattttgcacgacagata<br>taatattcacacattaccttgtgcgacggacgagccttaatctgtgggaatttgaactccgtgtggtttggattcatgAA<br>GCTTGGCACTGGCCGTCGTTTTACAACGTCGTGACTGGGAAAACCCTGGC<br>GTTACCCAACTTAATCGCCTTGCAGCACATCCCCCTTTCGCCAGCTGGCGT<br>AATAGCGAAGAGGCCCGCACCGATCGCCCTTCCCAACAGTTGCGCAGCCT<br>GAATGGCGAATGCTAGAGCAGCTTGAGCTTGGATCAGATTGTCGTTTCCC<br>GCCTTCAGTTTAAACTATCAGTGTTTGACAGGATATATTGGCGGGTAAAC

SUPPLEMENTARY METHODS

A step-by-step TTLOC protocol

Materials:

(1)Tn5 transposase, home-made, or commercially available (e.g.Novoprotein, Cat.M045)

(2) High-fidelity DNA polymerase enzyme for NGS (e.g. 2×TransStart® FastPfu Fly PCR SuperMix, TransGen Biotech, AS231)

(3) SPRI DNA purification beads

(4) Stock solution:

50% PEG8000

1M $MgCl_2$

1 M Tris(pH 8.0)

100 mM ATP

10% SDS

5× Tn5 buffer： 50 µL 1 M Tris(pH 8.0) ,20 µL 1 M $MgCl_2$, and 925 µL ddH_2O.

Annealing buffer:

(5) Oligos:

ME	AGATGTGTATAAGAGACAG	generating adaptors for Tn5 assembly
ME-rev	CTGTCTCTTATACACATCT(5'-Phosphate, 3'-AminolinkerC7)	generating adaptors for Tn5 assembly
ME-A	TCGTCGGCAGCGTCAGATGTGTATAAGAGACAG	generating adaptors for Tn5 assembly
N501	AATGATACGGCGACCACCGAGATCTACACTAGATCGCTCGTCGGCAGCGTC	NGS library P5 index primer
N502	AATGATACGGCGACCACCGAGATCTACACCTCTCTATTCGTCGGCAGCGTC	NGS library P5 index primer
N503	AATGATACGGCGACCACCGAGATCTACACTATCCTCTTCGTCGGCAGCGTC	NGS library P5 index primer
N504	AATGATACGGCGACCACCGAGATCTACACAGAGTAGATCGTCGGCAGCGTC	NGS library P5 index primer
N505	AATGATACGGCGACCACCGAGATCTACACGTAAGGAGTCGTCGGCAGCGTC	NGS library P5 index primer
N506	AATGATACGGCGACCACCGAGATCTACACACTGCATATCGTCGGCAGCGTC	NGS library P5 index primer
N507	AATGATACGGCGACCACCGAGATCTACACAAGGAGTATCGTCGGCAGCGTC	NGS library P5 index primer
N508	AATGATACGGCGACCACCGAGATCTACACCTAAGCCTTCGTCGGCAGCGTC	NGS library P5 index primer
N701	CAAGCAGAAGACGGCATACGAGATTAAGGCGAGTCTCGTGGGCTCGG	NGS library P7 index primer
N702	CAAGCAGAAGACGGCATACGAGATCGTACTAG	NGS library P7 index primer

	GTCTCGTGGGCTCGG	
N703	CAAGCAGAAGACGGCATACGAGATAGGCAGA AGTCTCGTGGGCTCGG	NGS library P7 index primer
N704	CAAGCAGAAGACGGCATACGAGATTCCTGAGC GTCTCGTGGGCTCGG	NGS library P7 index primer
N705	CAAGCAGAAGACGGCATACGAGATGGACTCCT GTCTCGTGGGCTCGG	NGS library P7 index primer
N706	CAAGCAGAAGACGGCATACGAGATTAGGCATG GTCTCGTGGGCTCGG	NGS library P7 index primer
N707	CAAGCAGAAGACGGCATACGAGATCTCTCTAC GTCTCGTGGGCTCGG	NGS library P7 index primer
N708	CAAGCAGAAGACGGCATACGAGATCAGAGAG GGTCTCGTGGGCTCGG	NGS library P7 index primer
N709	CAAGCAGAAGACGGCATACGAGATGCTACGCT GTCTCGTGGGCTCGG	NGS library P7 index primer
N710	CAAGCAGAAGACGGCATACGAGATCGAGGCTG GTCTCGTGGGCTCGG	NGS library P7 index primer
N711	CAAGCAGAAGACGGCATACGAGATAAGAGGC AGTCTCGTGGGCTCGG	NGS library P7 index primer
N712	CAAGCAGAAGACGGCATACGAGATGTAGAGG AGTCTCGTGGGCTCGG	NGS library P7 index primer
103LB2	GTCTCGTGGGCTCGGAGATGTGTATAAGAGAC AGGTGGCTTTATCTGTCTTTGTATTGT	pEarleyGate 103 LB region specific primer with P7 bridge sequence, working as LB bridge primer
103RB2	GTCTCGTGGGCTCGGAGATGTGTATAAGAGAC AGGCGTAATAGCGAAGAGGCCCGCA	pEarleyGate 103 RB region specific primer with P7 bridge sequence, working as RB bridge primer

Tn5 assembly(2.5 h) (Steps 1–4)

1. Add annealing buffer to oligos ME, ME-rev, and ME-A to make a 100 µM stock solution for each oligo.

2. Set up the following two reactions in PCR tubes:

Reaction 1 (ME/ME-rev): For a total volume of 20 µL, add 10 µL of 100 µM ME-rev and 10 µL of 100 µM ME-A.

Reaction 2 (ME-A/ME-rev): For a total volume of 20 µL, add 10 µL of 100 µM ME-rev and 10 µL of 100 µM ME-A.

Anneal the oligos using the program in the PCR machine (95°C for 3 min, 75°C for 15 min, 60°C for 10 min, 50°C for 10 min, 40°C for 10 min, 25°C for 30 min).

3. Mix the ME/ME-rev and ME-A/ME-rev at a 1:1 ratio, designated as the adapter mix(50 µM).

4. Set up the following reaction to generate the transposase: For a total volume of 6.2 μL, add 5 μL of Tn5 transposase (10 pmol/μL), 1.2 μL of adapter mix, pipette gently 20 times and mix well, incubated at 30°C for 1 hour. The final concentration of Tn5 transposase product is 8 pmol/μL. Store the transposase at -20°C.

TTLOC library construction and NGS (Steps 5–10)
Tagmentation:
5. Adjust genomic DNA of transgenic plants to a concentration of 200 ng/μL.
6. Set up the following reaction for each sample:

5× Tn5 buffer	4 μL
50% PEG	3.2 μL
ddH_2O	11.1 μL
Tn5	0.5 μL (4 pmol)
ATP	0.2 μL
Genomic DNA	1 μL (200 ng)

7. 55°C for 15 min in the PCR machine, then add 1 μL 1% SDS (20 μL 10% SDS to 200 μL ddH_2O) to stop the reaction, 72°C for 10 min in the PCR machine.

PCR amplification:
8. Set up the following PCR reaction (25 μL):

	Reaction 1	Reaction 2
Products from step 3	5 μL	5 μL
N50X（10 nM）	2 μL	2 μL
N70X（10 nM）	2 μL	2 μL
LB/RB Bridge primer (1 nM)	2 μL	2 μL
2×TransStart® FastPfu Fly PCR SuperMix	12.5 μL	12.5 μL
ddH_2O	1.5 μL	1.5 μL

Note: For each sample, at least one barcoding primer (N50X or N70X) need to be unique for dividing the NGS data.

PCR program:

Temperature	Time	Cycle
72°C	10 min	1
95°C	3 min	1
95°C	30 s	28–30
58°C	30 s	
72°C	30 s	
72°C	5 min	1

16°C	5 min	1

After PCR reaction, take 3 μL products for Gel electrophoresis analysis.

Library pooling:
9. Mix 10 μL products each from 6-12 reactions in step 8 together, added 1/2 volume SPRI beads to purify the DNA. Dissolve the DNA in 30–40 μL ddH_2O.

NGS:
10. Perform paired-end Illumina sequencing using an Illumina HiSeq 2500 or another massively parallel DNA sequencer, following the manufacturer's instructions. Obtain a 1-2 G raw data/library.

Data analysis (Steps 11–14)
11.Refer to the instruction in https://github.com/ShouliFeng2020/TTLOC, set up the environment and softwares needed for TTLOC.
12.Merge genome reference and tDNA reference.
Note: The name of tDNA reference sequence shoud be: "tDNA".

Usage:cat genome.fa tDNA.fa > genome.tDNA.fa

13. Built index for Merged genome
Usage: bwa index genome.fa tDNA.fa
14. Identify the T-DNA integration sites.
Usage: perl TTLOC.pl --genome /path/to/genome.tDNA.fa -1 input_1.fq -2 input_2.fq --prefix sampleID --tDNA /path/to/tDNA.fa
Note: refer to https://github.com/ShouliFeng2020/TTLOC for the descriptions of output files including "prefix".tDNA.summary, "prefix".displit.extra.fa, "prefix"_flank_seq.fa and "prefix".displit.extraVSGenome.txt.

PCR validation (Steps 15–17) (Optional)

15. Check the output files from step 14, design PCR primer pairs according to Figure 3b for TISs confirmation.
Note: A PCR primer pairs including a primer specific to flanking genome sequences of each TIS and a common primer specific to LB or RB region. The PCR products are generally 350-700 bp in size.
16. Perform the PCR reaction using genomic DNA of transgenic plants and the primer pairs designed in step 15. After PCR reaction, take 3 μL products for gel electrophoresis analysis.
17. Perform Sanger sequencing of the PCR products.
Note: For convenience,we recommend using corresponding common primer specific to LB or RB region used in step 16.
18. Perform the BLAST analysis using Sanger sequencing results as query and plant genome as reference, for the final confirmation of TISs.
